# Nationwide Estimate of Volatile Per- and Polyfluoroalkyl Substance (PFAS) Emissions from U.S. Landfills via Landfill Gas

**DOI:** 10.1021/acs.est.5c08763

**Published:** 2025-11-17

**Authors:** Florentino B. De la Cruz, Ivan A. Titaley, Yixuan Wang, Jennifer A. Field, Morton A. Barlaz

**Affiliations:** † Department of Civil, Construction, and Environmental Engineering, 6798 North Carolina State University, 915 Partners Way, Raleigh, North Carolina 27695, United States; ‡ Department of Environmental and Molecular Toxicology, 2694 Oregon State University, 1007 Agriculture and Life Sciences Building, Corvallis, Oregon 97331, United States

**Keywords:** volatile PFAS, landfill gas, FTOH, emissions, side chain fluoropolymer, TD-GC-MS, thermal desorption

## Abstract

Estimates of annual per- and polyfluoroalkyl substances (PFAS) emissions from landfill gas (LFG) using data from a nationwide sampling campaign have not been reported. The objective of this study was to characterize volatile PFAS in LFG and to estimate the annual mass of volatile PFAS released from US landfills. LFG samples were collected from 30 landfills in 17 states represented by different annual precipitation regions. PFAS concentrations varied by orders of magnitude, with a median concentration of 19,000 *ng*/*m* ^3^. Fluorotelomer alcohols, 6:2 and 8:2 FTOH, are the dominant PFAS in LFG, accounting for more than 95% of the total PFAS concentration. Minor components such as fluorotelomer olefins (FTOs) are also present, with concentrations ranging from 0–28,000 *ng*/*m* ^3^ and a median of 316 *ng*/*m* ^3^. The variability in PFAS concentrations was influenced by precipitation, as well as temporal and site-specific factors. By coupling the median concentration of PFAS with an estimate of US LFG emissions, it is estimated that 836 *kg*/*yr* of volatile PFAS are emitted from US landfills in uncollected gas with a 95% confidence interval (2.5% to 97.5% of the distribution) of 15–5,590 *kg*/*yr*. This estimate is comparable to ∼600 *kg* of PFAS released annually into landfill leachate.

## Graphical abstract

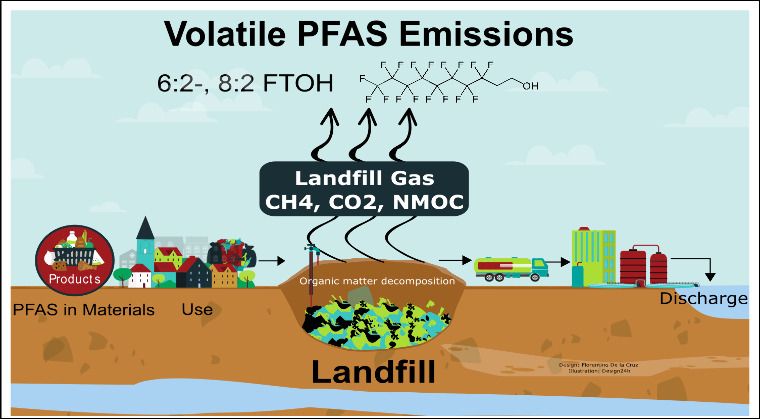

## Introduction

Per- and polyfluoroalkyl substances (PFAS) are a group of more than 4,000 anthropogenic chemicals that have been used in a wide range of industrial and consumer applications due to their unique chemical and physical properties, including their ability to repel water, oil, and stains.^1–3^ PFAS have been linked to various health and environmental concerns, leading to increased scrutiny and regulation.^2,3^ Landfills are a final disposal option for a wide range of consumer and industrial wastes, including materials containing PFAS, such as food packaging, stain-resistant fabrics, and carpets.^4–8^ In 2018, about 50% of the 265 Mt (million tons) of municipal solid waste (MSW) generated in the US was disposed of in landfills.^9^ Landfills are biologically active systems in which organic materials decompose predominantly through anaerobic pathways.^10^ Materials disposed of in a landfill undergo and/or are subjected to various chemical and biological processes that lead to the transfer of PFAS and other contaminants from solid waste to the liquid phase, as the water from precipitation percolates through the waste mass.^11,12^ As some PFAS are volatile, they have also been measured in LFG.

The presence of PFAS in landfill leachate is well documented.^8,13–18^ Landfill leachate characterization data are supported by laboratory studies showing the release of PFAS from carpet, textiles, food packaging, and residential MSW during anaerobic waste decomposition.^7,8,19^ Based on the analysis of leachate from 18 landfills, it was estimated that 600 kg of PFAS are released into the leachate annually.^13^ This leachate is typically sent to wastewater treatment plants where PFAS removal is limited, resulting in the release of PFAS into the environment through effluent water or biosolids.^20^

In contrast to landfill leachate, there is limited data on the presence and emissions of PFAS from landfill gas (LFG). To date, only three studies have measured PFAS directly in LFG. Goukeh et al.^21^ detected 6:2- and 8:2 fluorotelomer alcohols (FTOH) in the main header of a Florida landfill, using solid-phase microextraction gas chromatography-mass spectrometry (GC-MS), with concentrations ranging from 6,000 to 10,000 *ng*/*m* ^3^. Similarly, Titaley et al.^22^ reported up to 3,200 and 3,000 *ng*/*m* ^3^ 6:2- and 8:2 FTOH respectively, in three landfills in the southeastern US, using thermal desorption - gas chromatography - mass spectrometry (TD-GC-MS). Minor components such as 12:2 fluorotelomer olefins (FTO) were also reported. Lin et al.^23^ used PUF/XAD-2 cartridges to sample a wellhead from three Florida landfills followed by methanol/ethyl acetate extractions and then GC-MS. FTOH concentrations were estimated (up to 170,000 and 740,000 *ng*/*m* ^3^ for 6:2- and 8:2 FTOH, respectively) as they exceeded the calibration curves. The sum of 6:2-, 8:2- and 10:2 FTOH represented 87 to 97% of the total PFAS in LFG.

Site- and national-level estimates of the contribution of volatile PFAS from LFG to the total mass release of PFAS from landfills were conducted using a mass balance approach. Based on data from three Florida landfills, Lin et al.^23^ estimated that LFG accounts for 32–76% of total fluorine leaving the landfill, which is comparable to or greater than the mass of fluorine leaving through the leachate pathway. At the national level, Tolaymat et al.^24^ calculated PFAS emissions through LFG using the concentrations reported by Titaley et al.^22^ and estimated that 500 kg/year (6% of total PFAS) will be emitted through LFG, compared to 800 kg/year (10% of total PFAS) through leachate. The study also estimated that about 6,300 kg/year or (84% of total PFAS) will accumulate in a landfill. Although prior studies are important in assessing the fate of PFAS, the authors acknowledged the limitations of their estimate, including limited data, particularly in spatial and temporal scope. Therefore, a comprehensive assessment of PFAS concentrations in LFG is essential to evaluate national emissions. These data are crucial for mapping the national and global mass flows of PFAS in the environment, which is fundamental for assessing related public health risks.

The objective of this study was to develop an estimate of PFAS emissions from US landfills through LFG. Thirty landfills were sampled in 17 states that include arid, moderate and wet precipitation regions to assess climatic effects. LFG was sampled up to four times at various locations to evaluate PFAS speciation and temporal variability in LFG. The effect of the downstream condensate removal process that is used before the combustion of LFG in a flare or internal combustion engine (ICE) was also evaluated. Samples were captured in multibed sorbent tubes and analyzed by TD-GC-MS. The representative concentration of PFAS in LFG that is not collected and therefore released to the atmosphere was coupled with an estimate of US LFG emissions to estimate PFAS emissions from US landfills.

## Materials and Methods

### Site Description and Sample Collection

LFG was sampled in 30 US landfills (Table S1). The landfills were classified as located in arid (<508 mm), moderate (508 −1016 mm) or wet (>1016 mm) climatic regions using the categories specified in the Greenhouse Gas Reporting Program (GHGRP).^26,27^ Site selection was based on availability, resulting in a higher number of wet sites (17) compared to sites in moderate (eight) and arid (five) regions (Table S1). As presented in the Results, the distribution of the sampled landfills was close to the distribution of US LFG generation. The study sites accepted primarily MSW with small amounts of construction and demolition (C&D) wastes and biosolids. To capture temporal variability, sampling was performed twice for 26 sites and four times for two sites. Two landfills were only sampled once. LFG was sampled from the main header, which is a composite of LFG collected from all areas of each landfill. Samples were collected upstream and downstream of the condensate knockout system to evaluate its impact on PFAS concentrations. Condensate knockout is used prior to combustion of LFG in a flare or ICE, and its impact on PFAS concentrations is important since not all PFAS that is burned is necessarily fully oxidized, as described below.

A sampling system was constructed to collect LFG from both individual gas wells and the main pipe (header) to analyze volatile PFAS by TD-GC-MS as previously described.^22^ The method was optimized for 28 target volatile PFAS in eight different groups (Table S2) and to detect 14 suspect PFAS (Table S3) from four classes listed in the Supporting Information (SI).^22^

Briefly, samples were collected using a system consisting of a sampling port connected to the LFG header pipe followed by a glass fiber filter and a three-position solenoid valve to direct flow toward a manifold where triplicate TD tubes were installed in parallel. A sample volume of 350 mL of LFG, determined from previous optimization experiments to avoid breakthrough, was obtained by passing 100 ± 0.1 mL/min through each of the three sorbent tubes positioned in parallel. A factory calibrated mass flow controller (Alicat Scientific, Tucson, AZ) was used to provide an accurate volume of gas. A GEM 5000 (LANDTEC, Dexter, MI) was also connected inline to measure methane concentrations, followed by a connection to an explosion-proof vacuum pump (KNF, Germany) to overcome the vacuum (−7 to −2 kPa) of the gas collection system. Flexible Tygon tubing was connected using stainless steel hose clamps, while rigid stainless steel tubing was connected with compression fittings. Following sampling, the TD tubes were capped, shipped to Oregon State University (OSU) at ambient temperature, and stored at 4 °*C* before analysis.

### Sample Analysis

#### Chemicals

The names, abbreviations and information for 28 target PFAS (including three secondary fluorotelomer alcohols, sFTOH) and 14 suspect PFAS, 10 mass-labeled surrogate standards and a mass-labeled internal standard are provided in Tables S2–S4. A mixture of 10 surrogate standards was prepared in methanol at 500 *pg*/*uL* each. The internal standard (Table S3) was prepared at 100 *pg*/*uL* in methanol. Additional information on standards is given in the SI.

#### Thermal Desorption-GC-MS

‘Universal’ stainless steel sorbent tubes consisting of a polymer, carbon black, and carbon black molecular sieve (Markes) were selected on the basis of prior TD-GC-MS reports.^22,28^ Prior to sampling, the TD tubes were spiked with 500 pg of the ten surrogate standards (Table S4), capped and stored at ≤ 4 °*C* before shipping (at ambient temperature) to North Carolina State University (NCSU) for use at landfill sites. Surrogate standard-spiked TD tubes were brought to room temperature and spiked with 100 pg internal standard.

A TD100-xr (Markes International) system was used to desorb the TD tubes for 12 min at 320 °C with (50 *mL* He/*min*). The flow path temperature was 220 °C and the tubes were dry purged for 1 min with 50 *mL* N_2_/*min*. Volatile PFAS was focused on a general-purpose sorbent-packed cold-trapping system. The desorption of the analytes from the cold trap occurred during a ramp from 25 to 335 °C at 100 °C/s and was held for 5 min prior to desorption and introduction of the sample into the GC inlet. GC separation and MS details, including quantifier and qualifier ions for target and suspect PFAS, surrogate, and internal standards are given in Tables S2–S4.

Calibration curves were developed for the volatile PFAS target by spiking 10 TD tubes with the mix of PFAS, ranging between 1 and 250,000 pg in a TD tube (Table S5). The concentrations of suspect volatile PFAS (Table S3) were estimated from the calibration curve of chemically similar target volatile PFAS (Table S2), assuming equimolar response factors.

#### Quality Assurance/Quality Control (QA/QC)

The trip blanks consisted of surrogate-spiked TD tubes shipped to a landfill, which remained unopened during sampling and then shipped back to OSU. Field blanks consisted of surrogate-spiked TD tubes shipped to a landfill, opened for a single LFG sampling event (e.g., 3.5 *min*) and shipped back to OSU.^22^ No statistically significant detection was observed in opened and unopened blanks. Before sample collection, the sampling train was flushed for 10 min to eliminate possible PFAS carryover. Instrumental limit of detection (iLOD) and limit of quantitation (LOQ) were previously reported.^22^ The LOD was calculated based on linear regression with weighting 1/*x* and *LOQ* = 3.3 × *LOD*. The LOQs were compared with the lowest calibration mass in the calibration curves; the higher (conservative) mass was selected as the LOQ and was divided by the final LFG sampling volume to calculate a whole LOQ LFG sampling method.^29^ Accuracy and precision of target PFAS analysis were determined based on the recovery and relative standard deviation (RSD) of three TD tubes spiked in the laboratory with 50 and 500 pg of target PFAS.^22^ Surrogate recovery for the TD tubes sent to the field was used to calculate accuracy and precision. On average, the coefficient of variation (CV) among triplicate samples collected at the same time is 26% (*CV* = *SD*/*mean* × 100%), which reflects the variability associated with sampling and analysis. Previously, analytical variability was reported to have CV of up to 28%.^22^ Samples with instrument response below the limit of detection (<LOD) and below *LOQ* were treated as zero to allow inclusion of these data points for quantitative analyses.

### Data Analysis and Model Development

The total PFAS (SumPFAS) was calculated as the sum of all PFAS concentrations, except sFTOH, since it was not consistently measured in all samples. Similarly, we calculated the sum of the concentrations for each PFAS group as *SumFTOH* = ∑(*n*: 2 *FTOH*), *SumFTO* = ∑(*n*: 2 *FTO*), *SumFOSA* = ∑(*N* – *MeFOSA*, *N* – *EtFOSA*), *SumFOSE* = ∑(*N* – *MeFOSE*, *N* – *EtFOSE*), *SumFTAc* = ∑(*n*: 2 *FTAc*), *SumFTMAc* = ∑(*n*: 2 *FTMAc*), *SumFTI* = ∑(*n*: 2 *FTI*). PFAS abbreviations are defined in the SI (Tables S2 and S3).

A model was developed to quantify the mass of PFAS released in US LFG (eq 1). Here, the model is described for SumPFAS, although it could be applied to individual compounds or compound groups. The model was written to maximize flexibility though its parametrization was simplified in consideration of available data as described below. The model considers the concentration of PFAS in landfill gas and allows the concentration to vary by climate. County-level precipitation data was used to group landfills into arid, moderate and wet climatic regions using the GHGRP criteria (Table S1).^26,27^ The model allows the PFAS concentration to vary upstream and downstream of the condensate knockout system of the landfill, although, as described below, there was no statistical difference in the concentration of SumPFAS up and downstream of the condensate knockout system in this study and only one set of concentrations was used as described in the Results. The volume of US LFG that is collected and the volume released as fugitive emissions are also incorporated into the model. The mass of PFAS in the collected gas (*SumPFAS* × *volume collected*) is then reduced by the destruction efficiency in the LFG treatment device (flare or ICE). Our base case assumption is 100% destruction of PFAS in the LFG treatment device, although we are unaware of any published supporting data and the destruction efficiency was reduced to 50% in a series of sensitivity analyses applied to the model (see Results). Finally, the mass of PFAS in uncollected LFG is calculated as the volume of fugitive emissions in US LFG multiplied by the SumPFAS in LFG. All analyses assume no attenuation of PFAS as it passes through the landfill’s cover soil although some PFAS attenuation could occur. Our limited data set did not suggest any attenuation of PFAS.^30^

The volume of US LFG collected and fugitive emissions were estimated using data from the US EPA Greenhouse Gas Inventory (USGHGI), GHGRP, and the US EPA Landfill Methane Outreach Program (LMOP) database as described in the SI.^26,27,31,32^ Here too there is uncertainty as presented in the SI and explored in the sensitivity analysis in the results. Finally, we acknowledge that the sumPFAS in collected LFG can vary from the concentration released as fugitive emissions because the collected gas is often influenced by air intrusion that dilutes PFAS concentrations. PFAS concentrations for use in eq 1 were corrected for air intrusion into the LFG collection system as described in AP-42 (eq S1).^33^ For our data set, this dilution effect is within 28%, which is comparable to or less than the uncertainties of other input parameters (e.g., flare and ICE destruction, LFG volumes, PFAS concentrations).

1 $$PFAS\;\mathrm{Emissions}=\underset{i\epsilon \left\{\mathrm{arid},\mathrm{moderate},wet\right\}}{\sum }C_{\mathrm{PFAS}u,i}V_{f,i}\times \left(1-AF\right)+C_{\mathrm{PFAS}d,i}\left[V_{\mathrm{flare},i}\times \left(1-DE_{\mathrm{flare}}\right)+V_{ICE,i}\times \left(1-DE_{ICE}\right)\right]$$

where *C*_*PFASu*,*i*_ and *C*_*PFASd*,*i*_ = SumPFAS in LFG in each precipitation class (*i*), upstream­(*u*), and downstream (*d*) of condensate knockout, respectively (*ng*/*m* ^3^); *V*_*f*,*i*_ = total volume of LFG that escapes through the soil cover (*Mm* ^3^); *AF* = PFAS soil attenuation factor (i.e., fraction of total PFAS attenuated in the soil cover); *V*_*flare*,*i*_ = volume of LFG treated in a flare (*Mm* ^3^); *V*_*ICE*,*i*_ = volume of LFG converted to energy in an ICE (*Mm* ^3^); and *DE*_*flare*_,*DE*_*ICE*_ = PFAS destruction efficiencies in LFG flare and ICE, respectively.

A Shapiro–Wilk normality test was performed to determine the appropriate test for significant differences between the sample groups. For the non-normal distributions, we used the Kruskal–Wallis and/or Mann–Whitney U statistical test as described in the SI, to determine the effect of different environmental and landfill characteristics. All statistical tests were conducted at a level of significance, α = 0.05. Data analyses were performed with R, Python, and MS Excel.

## Results and Discussion

### Occurrence of PFAS in Landfill Gas from US Landfills

Paired comparison of non-normal distributions (Figure S1) of SumPFAS upstream and downstream of the condensate knockout system (Table S6) using the Kruskal–Wallis and Mann–Whitney U test was performed to assess whether condensate removal at the main header contributes to PFAS removal. Neither test revealed a significant difference between the SumPFAS upstream and downstream (*p* > 0.05), prompting a combination of upstream and downstream data for subsequent analyses (Table 1). This observation is in contrast to an earlier study by Smallwood et al. in which LFG condensate collected from a sump was dominated by volatile PFAS 5:2- and 7:2 sFTOH, indicating that these compounds are likely to partition into the aqueous phase and are removed from the gas phase.^34^ The primary difference between the work by Smallwood^34^ and the current study is the sampling location. The first stages of LFG moisture removal occur in condensate sumps distributed throughout the well field. Consequently, when the LFG arrives at the condensate knockout in the main header, it is likely that most of the moisture containing volatile PFAS has been removed such that no significant PFAS removal was observed in the LFG in the main header.

**Table 1:** Volatile PFAS Detected in LFG Samples Collected from 30 Landfills across 17 States in the US

GHGRP Class^a^	Alias	Status	Age (yr)^b^	ppt (mm)^c^	n^d^	PFAS Detected^e^	Median SumPFAS $\left(\frac{\mathrm{ng}}{m^{3}}\right)$	Mean SumPFAS $\left(\frac{\mathrm{ng}}{m^{3}}\right)$	SD $\left(\frac{\mathrm{ng}}{m^{3}}\right)$ ^f^	Dominant PFAS^g^
Wet	LF1	Open	49	1228	11	9	9.24 × 10^3^	2.25 × 10^4^	2.48 × 10^4^	6:2 FTOH (87%)
Wet	LF11	Open	69	1064	11	6	6.00 × 10^4^	3.99 × 10^4^	3.42 × 10^4^	10:2 FTOH (54%)
Wet	LF12	Open	30	1341	9	7	2.87 × 10^4^	1.88 × 10^4^	1.54 × 10^4^	6:2 FTOH (89%)
Wet	LF13	Open	16	1315	11	7	3.28 × 10^3^	1.63 × 10^4^	1.64 × 10^4^	6:2 FTOH (59%)
Wet	LF15	Open	46	1228	6	4	8.02 × 10^4^	9.09 × 10^4^	1.02 × 10^5^	4:2 FTOH (100%)
Wet	LF16	Open	43	1302	15	7	2.31 × 10^3^	2.21 × 10^4^	2.96 × 10^4^	10:2 FTOH (57%)
Wet	LF18	Open	41	1413	12	5	1.29 × 10^4^	1.74 × 10^4^	1.88 × 10^4^	8:2 FTOH (94%)
Wet	LF19	Open	42	1463	22	8	7.96 × 10^4^	9.79 × 10^4^	8.88 × 10^4^	10:2 FTOH (66%)
Wet	LF2	Open	21	1153	13	7	1.89 × 10^4^	3.24 × 10^4^	2.41 × 10^4^	6:2 FTOH (96%)
Wet	LF21	Open	70	1648	6	9	6.92 × 10^3^	6.81 × 10^3^	1.16 × 10^3^	10:2 FTOH (65%)
Wet	LF23	Closed	35	1183	12	5	9.25 × 10^3^	2.67 × 10^4^	3.56 × 10^4^	6:2 FTOH (63%)
Wet	LF24	Open	43	1590	11	7	3.08 × 10^4^	3.74 × 10^4^	2.04 × 10^4^	6:2 FTOH (64%)
Wet	LF26	Open	13	1176	25	6	8.77 × 10^3^	1.24 × 10^4^	1.01 × 10^4^	6:2 FTOH (67%)
Wet	LF28	Open	21	1369	12	5	1.37 × 10^4^	1.97 × 10^4^	2.26 × 10^4^	12:2 FTO (71%)
Wet	LF29	Open	48	1079	4	9	3.55 × 10^4^	3.80 × 10^4^	5.78 × 10^3^	6:2 FTOH (94%)
Wet	LF30	Open	25	1249	3	7	3.09 × 10^4^	2.85 × 10^4^	5.52 × 10^3^	6:2 FTOH (84%)
Wet	LF4	Open	44	1262	12	6	2.64 × 10^3^	4.15 × 10^3^	3.74 × 10^3^	6:2 FTOH (72%)
Moderate	LF10	Open	43	1006	12	8	2.48 × 10^4^	4.08 × 10^4^	4.42 × 10^4^	10:2 FTOH (63%)
Moderate	LF14	Closed	76	1001	15	5	3.40 × 10^3^	3.12 × 10^3^	2.63 × 10^3^	10:2 FTOH (77%)
Moderate	LF17	Open	40	837	9	5	2.78 × 10^4^	2.15 × 10^4^	1.48 × 10^4^	6:2 FTOH (86%)
Moderate	LF20	Open	45	1004	12	8	1.87 × 10^5^	1.94 × 10^5^	4.59 × 10^4^	6:2 FTOH (66%)
Moderate	LF22	Closed	44	979	11	4	2.09 × 10^3^	1.92 × 10^3^	1.12 × 10^3^	10:2 FTOH (70%)
Moderate	LF25	Open	39	887	11	8	1.01 × 10^5^	8.20 × 10^4^	6.30 × 10^4^	6:2 FTOH (87%)
Moderate	LF6	Open	48	923	11	7	8.81 × 10^4^	8.97 × 10^4^	2.13 × 10^4^	6:2 FTOH (71%)
Moderate	LF8	Open	44	812	10	7	8.79 × 10^4^	9.18 × 10^4^	2.43 × 10^4^	6:2 FTOH (60%)
Arid	LF27	Open	44	391	10	6	3.00 × 10^3^	2.72 × 10^3^	8.58 × 10^2^	12:2 FTO (68%)
Arid	LF3	Open	47	327	6	7	4.52 × 10^3^	4.57 × 10^3^	4.62 × 10^2^	10:2 FTOH (53%)
Arid	LF5	Open	44	290	11	7	4.58 × 10^4^	7.39 × 10^4^	5.14 × 10^4^	8:2 FTOH (95%)
Arid	LF7	Open	51	226	10	8	9.19 × 10^4^	8.61 × 10^4^	3.72 × 10^4^	4:2 FTOH (100%)
Arid	LF9	Open	31	443	11	9	1.76 × 10^5^	1.70 × 10^5^	4.19 × 10^4^	6:2 FTOH (70%)

a GHGRP precipitation classification based on refs 26 and 27 .

b Landfill age calculated as the difference between the sampling date and the date the landfill opened.

c NOAA Annual normal precipitation at the closest weather station.^35^

d Number of samples successfully analyzed. Three to seven replicate samples were collected in each sampling event with each of the triplicate TD tubes in the sampling train treated as a unique sample. Of the 30 landfill sites, two were sampled four times, 26 were sampled twice, LF29 and LF30 were sampled once.

e Mean number of PFAS detected.

f SD – Standard deviation.

g Dominant PFAS and its corresponding percentage of the SumPFAS. Calculated based on the median value.

For the 30 landfills, the median SumPFAS was 19,000 *ng*/*m* ^3^, with an average of 47,300 *ng*/*m* ^3^ (standard deviation SD = 60,700 *ng*/*m* ^3^). Notable differences between landfills are evident (Table 1 and Figure 1) with LF20 (moderate), LF9 (arid) and LF25 (moderate) showing the highest median SumPFAS at 1.87 × 10^5^ *ng*/*m* ^3^, 1.76 × 10^5^ *ng*/*m* ^3^ and 1.01 × 10^5^ *ng*/*m* ^3^, respectively. Closed landfills in general have lower SumPFAS compared to open landfills (Figure S2). Whether this observation can be generalized given the limited data set is uncertain. The SumPFAS ranges are comparable to previously reported values.^21–23^

**Figure 1:**
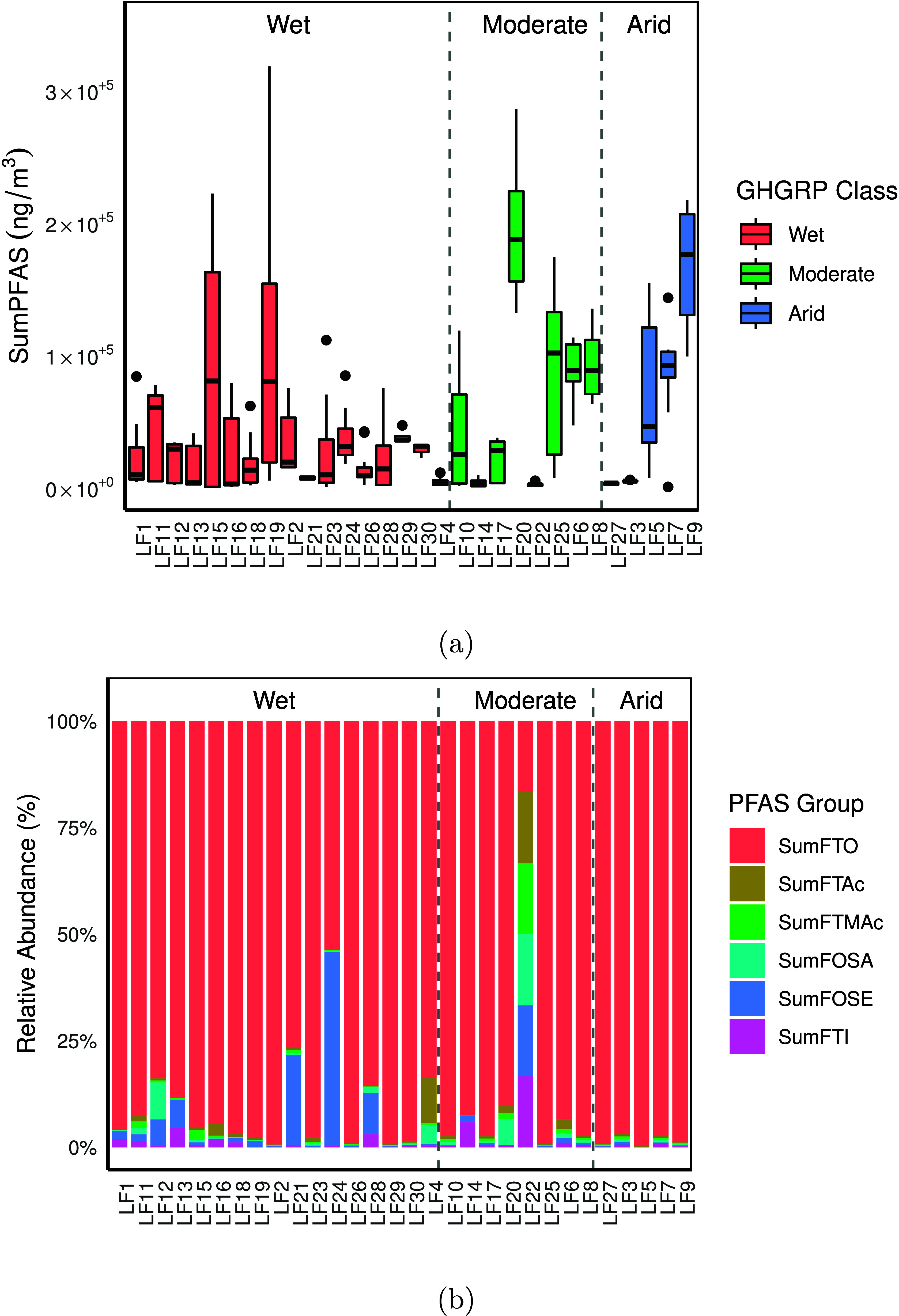
(a) Ranges of SumPFAS in 30 US landfills grouped by precipitation region as defined by the GHGRP.^26^ The boxes bound the interquartile range (IQR) (25th to 75th quartile). The whiskers extend to 1.5 times the IQR, and their ends show the highest and lowest value excluding outliers, which are represented by points. (b) Relative composition calculated based on the mean of the sum of minor PFAS groups excluding FTOH, which accounts for the majority of volatile PFAS in LFG with a median value of 98%. Only FTO was consistently detected in all landfills.

The empirical density distribution for SumPFAS concentrations in 30 US landfills indicates substantial variability (Figure 2). The distribution of SumPFAS is skewed to the right and is characterized by overdispersion. One possible explanation for the elevated levels of PFAS depicted in the tail of the distribution is that these landfills accepted wastes that contained PFAS, such as contaminated soil from firefighting pits, spent water treatment residuals, and autoshredder residue. Higher concentrations of PFAS in leachate from landfills that accept a range of PFAS-laden wastes, as compared to landfills that accept MSW only, have recently been reported.^36^ It is unknown whether sites that accept PFAS-laden wastes would contain elevated concentrations of PFAS in the gas. However, other factors also influence PFAS concentrations in LFG such as the age of PFAS-containing packaging materials and the LFG flow rate.^19^

**Figure 2:**
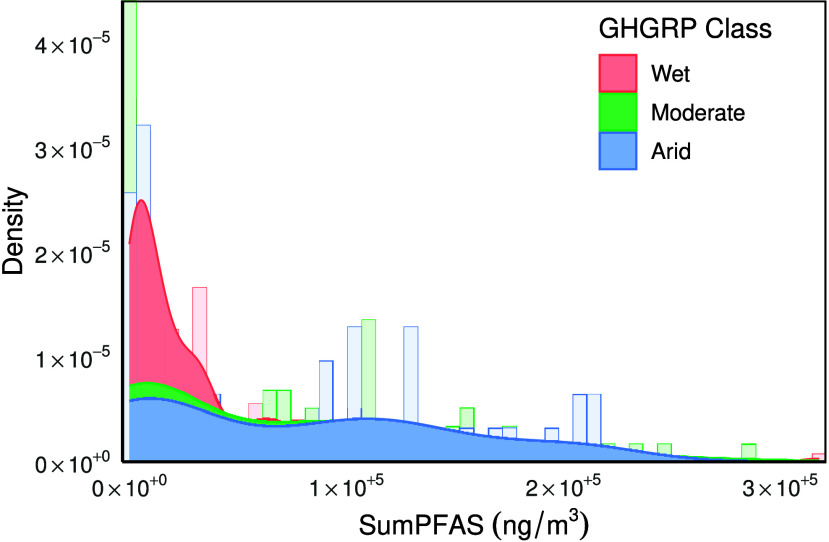
Distribution of SumPFAS across 30 US Landfills: Histogram (bars, 50 bins) with Kernel Density Estimates (smooth lines) by precipitation class (Wet: *n* = 195, Moderate: *n* = 48, Arid: *n* = 48). The *y*-axis shows relative frequency. SumPFAS was statistically similar between arid and moderate landfills and greater than the concentrations in wet landfills.

Table 2 shows the SumPFAS point estimates derived from the PFAS concentration data for input to the PFAS emissions model (eq 1). It is interesting to note that while extremely high concentrations were observed in some samples, more than 25% of SumPFAS in the distribution have concentrations below 10,000 *ng*/*m* ^3^ (Table 2). Given the substantial variability in SumPFAS and the observation that a few landfills have concentrations well above the median, it is likely that a few landfills may have a large influence on the estimate of US PFAS emissions in LFG as presented below.

**Table 2:** Distribution of SumPFAS in LFG (*ng*/*m* ^3^)­^a^

GHGRP Class^b^	2.50%	25%	50% (median)	75%	97.50%
Wet	5.77 × 10^1^	5.05 × 10^3^	1.82 × 10^4^	4.95 × 10^4^	2.32 × 10^5^
Moderate	9.24 × 10^2^	3.08 × 10^3^	4.27 × 10^4^	1.35 × 10^5^	2.69 × 10^5^
Arid	2.21 × 10^3^	5.12 × 10^3^	8.93 × 10^4^	1.41 × 10^5^	2.34 × 10^5^

a SumPFAS concentrations upstream and downstream of condensate knockout were statistically similar (*p* > 0.05) and thus combined.

b Even though arid and moderate climates had similar PFAS levels (no significant difference), we kept them separate in the emissions calculations to retain potential climate-specific differences in LFG generation (see Table S13).

#### Diversity of Volatile PFAS in LFG

Among the 42 target and suspect PFAS analyzed, 28 compounds were identified. Each sample contained 4 to 9 different PFAS (Tables 1 and S8). FTOHs make up the majority of PFAS, with a median value of 98% of SumPFAS and a range of 27–100%. FTOH homologue 6:2 FTOH was the highest fraction in most samples, representing a median of 50% of SumPFAS, followed by 8:2 FTOH with a median of 13.6% (Table 1).

Characterized by a perfluorinated carbon chain with an alcohol end group, FTOH is synthesized by a telomerization process to produce homologues of the target compound.^3^ FTOHs are used as building blocks for the production of polymeric PFAS including telomer-based side chain fluoropolymers (SCFP) and fluorosurfactants with applications including household cleaning agents, paints, carpet and paper coatings.^2,37–39^ Fluorotelomer production is composed of 80% polymer and 20% surfactants, and it is estimated that production related to the side chain of the 8:2 FTOH polymer was 2,145 tonnes in 2004.^40^ FTOH is present in consumer products, particularly food packaging,^41^ which, at the end of its useful life, is generally disposed of in landfills. Until its voluntary ban in the US in 2020, FTOHs were commonly used in grease-resistant materials used for food contact applications, such as paper and paperboard food packaging.^42^

There are two likely mechanisms for the realease of FTOHs to LFG. First, FTOH may be released from the “off-gassing” of unreacted FTOH, presumably due to incomplete reaction and lack of downstream purification. Dinglasan-Panlilio and Mabury estimated that for the fluorinated materials they tested, the residual alcohols consisted of 0.04–3.8% based on a fluoro alcohol to dry mass basis.^37^ Second, FTOH can be released as a result of biotic and abiotic transformations of SCFP such as the fluoroacrylate polymer where the fluorotelomer side chain is linked by a covalent bond (e.g., ester linkage). Hydrolysis of this linkage can result in the release of FTOH. This reaction was demonstrated in fluoroacrylate polymer incubated in aerobic soil, where the degradation of the polymer released FTOH with terminal reaction products, including perfluorocarboxylic acids.^43^

Secondary FTOHs (sFTOH) were also measured in selected landfills but were not included in the total because they were not measured at all sites (Table S7). For the samples where sFTOH was measured, the range was similar to that previously reported.^23^ The exclusion of sFTOH from SumPFAS is likely not a significant limitation because on average sFTOHs make up 2.4% of SumPFAS (Table S7). It is not surprising that sFTOHs are detected in LFG, as sFTOHs are intermediates in the oxidation of FTOH. For example, when 8:2 FTOH undergoes oxidation, it forms the telomer aldehyde 8:2 FTAL, which then undergoes further oxidation at the terminal carbon, forming 8:2 FTCA. This 8:2 FTCA can further transform into its unsaturated form, 8:2 FTUCA, and ultimately to 7:2 sFTOH before transforming into PFOA.^37,44–47^ A correlation heatmap presented in Figure S3 shows that the 5:2 sFTOH is highly correlated with 6:2, 8:2 and 10:2 FTOH, and 3:2 sFTOH is strongly correlated with 4:2 FTOH (*r* = 0.55, Figure S3).

We also detected minor components such as FTO, particularly 12:2 FTO (Figure 1b, Table S8). FTOs are precursors to the production of FTOH, therefore, it is not surprising to detect FTO in LFG^2,3,48^ and its presence aligns with previous reports.^22,23^ It is surprising that 12:2 FTO was the dominant volatile PFAS in LF 27 and 28 (Table 1), suggesting a large abundance of residual FTOH precursor relative to the other landfills sampled. Although the number of PFAS in LFG was generally limited to 4 to 9 compounds, the concentrations varied by orders of magnitude (Tables 1 and S8).

### Factors Affecting the Presence of Volatile PFAS in Landfill Gas from US Landfills

Figure S4 shows the correlations between the different PFAS and site-specific conditions such as landfill age, LFG composition, and precipitation. The utility of Figure S4 is demonstrated by a strong positive correlation between CH_4_ and CO_2_ (*r* = 0.89). The strong correlation is expected, as most of the degradable organic carbon in landfills is attributed to lignocellulose.^49^ The decomposition of lignocellulosic materials in landfills results in the production of equimolar CH_4_ and CO_2_.

The strong correlation of SumPFAS with FTOH demonstrates the dominance of FTOH in total PFAS concentrations. With respect to individual compounds, 8:2 FTOH was strongly positively correlated with 6:2 FTOH (r = 0.79) and 12:2 FTOH was positively correlated with n:2 FTO which warrants future investigation.

The homologues of FTOs are positively correlated with each other. For example, the 10:2 and 8:2 FTO correlation coefficient is 0.95. As noted above, FTOs are precursors to the production of FTOHs.

There was no strong correlation between SumPFAS and age, CH_4_ composition, and precipitation (Figure S4). However, the pairplot of PFAS with other variables (Figure S5) reveals that PFAS detection is higher in landfills with a higher methane composition, indicating that PFAS release is correlated with waste decomposition. The lower composition of CH_4_ is typically observed in the early and later phases of MSW decomposition, and ≥ 50% marks the onset of the methanogenic phase where the observed SumPFAS are the highest. However, there are many operational factors that influence the concentration of CH_4_ in LFG.

PFAS concentrations were the highest in landfills with ages of 30–60 years compared to PFAS concentrations at <30 years and >60 years. This observation is contrary to a recent laboratory-scale study in which PFAS were released early in the decomposition cycle.^19^ This difference is perhaps due to the timing of waste deposition. In a lab-scale batch reactor, waste is deposited at a single time point, and waste decomposes at the same time. In a field-scale landfill, waste is continuously deposited, resulting in waste with varying states of decomposition.

Nonparametric Kruskal–Wallis and Mann–Whitney U pairwise statistical tests on the distribution of SumPFAS in wet, moderate, and arid landfills (Figure 2) suggest that arid and moderate landfills were statistically similar and SumPFAS was higher than in wet landfills. A plausible explanation for this observation is a “flushing effect”. Landfills with higher precipitation generate more LFG (Table S14). Thus, assuming similar waste and PFAS composition in landfills in wet, moderate and arid climates, more gas is flushing the same mass of released PFAS, resulting in lower PFAS concentrations in LFG in wet climates. In addition, more gas generation from landfills in wet climates would release PFAS earlier, when a gas collection and control system might not be in place. This is particularly true for FTOHs, where one mechanism of release is “off-gassing,” as discussed above.^50^ In addition to being volatile, the decomposition of cellulosic materials such as paper to which FTOHs adhere would promote PFAS release. Lower gas production coupled with an accumulation of PFAS released from volatilization likely resulted in higher PFAS concentrations in arid and moderate landfills.

The CV of the samples collected at the same locations in the landfill on different dates is 69%, which reflects temporal variability. In general, the PFAS detected on different sampling dates was comparable, but the concentrations varied by up to an order of magnitude. For the two landfills sampled four times, the SumPFAS CVs were 93% (LF19) and 79% (LF26).

### Estimate of PFAS from LFG As Production and As Emission

The SumPFAS summarized in Table 2 are coupled with estimates of the annual volume of LFG that is treated by a flare or ICE [2.18 × 10^4^ million *m* ^3^ (*Mm* ^3^)] and LFG that is released as fugitive emission (2.30 × 10^4^ *Mm* ^3^) to develop an estimate of US gaseous PFAS emissions attributed to LFG. The annual volumes of LFG that are treated or released as fugitive emissions are presented in Table S14 and the accompanying text. Several scenarios are explored in the sensitivity analysis below.

The SumPFAS concentrations used to estimate the total US release are presented in Table 2, which includes values that represent 2.5, 25, 50 (median) 75 and 97. 5% of the distribution of the likely concentrations in arid, moderate and wet climates. A series of cases was used to capture the range of plausible SumPFAS emissions (Table 3). In the base case (Case 1), we used the median SumPFAS by climate and assumed 100% destruction of PFAS in an ICE and flare. No attenuation of PFAS in the landfill cover soil was assumed for any case. As described earlier, there are no data to support the attenuation of PFAS in cover soil. Case 1 results in US LFG PFAS emissions of 836 *kg*/*yr*.

**Table 3:** Estimates of PFAS Emissions for a Series of Plausible Scenarios^a^

Case	PFAS Emissions (kg/yr)	PFAS Destruction in flare or ICE (%)	Description
Case 1: Base Case	836	100	Median PFAS concentrations from Table 2.
Case 2: 25–75% quartiles	103–2,030	100	Use the 25–75% quartile PFAS concentrations from Table 2.
Case 3: 2.5–97.5% quantiles	15–5,590	100	Use the 2.5–97.5% quantile PFAS concentrations from Table 2.
Case 4: Incomplete PFAS Combustion	1,240	50	Assume 50% PFAS destruction in flares and engines; median PFAS concentrations from Table 2.
Case 5: Lower estimate of fugitive emissions	398	100	Use the lower estimate of LFG fugitive emissions calculated as described in Table S14; median PFAS concentrations from Table 2.
Case 6: 25–75% quartiles with lower fugitive emissions estimate	47–974	100	Use the lower estimate of LFG fugitive emissions calculated as described in Table S14 and 25–75% quartile PFAS concentrations from Table 2.
Case 7: Lower estimate of fugitive emissions and incomplete combustion	807	50	Use the lower estimate of LFG fugitive emissions calculated as described in Table S14, median PFAS concentrations from Table 2, and 50% PFAS destruction in flares and engines.

a PFAS soil attenuation factor (AF) was assumed to be 0 for all cases due to the lack of data supporting any PFAS attenuation in soils.

The first set of sensitivity analyses considers the emission range based on the measured range of SumPFAS (Cases 2 and 3). PFAS emissions based on the concentration of the 75% quartile are 20 times higher than the 25% quartile, and this ratio increases to 372 when considering the 2.5 and 97.5% quantiles.

The total volume of LFG that is treated in a flare plus an ICE is similar. Thus, a low PFAS destruction efficiency in a control device (flare, ICE) will measurably increase PFAS emissions. Unfortunately, there are no data on the PFAS destruction efficiency in either an ICE or a flare. While the base case (Case 1) assumed 100% PFAS destruction, a case with 50% destruction efficiency for both a flare and an ICE increases emissions by approximately 50% relative to the base case (Case 4).

There are two methods that are used in the GHGRP to estimate fugitive emissions from landfills. Our base case used the higher estimate of 2.3 × 10^10^ *m* ^3^, which is the average of the upper and lower estimates in Table S14. The alternate methodology results in an estimate of 1.07 × 10^10^ *m* ^3^ and the corresponding point estimate of PFAS emissions decreases in direct proportion to fugitive emissions (Case 5). The discrepancy in the volume of fugitive emissions has been recognized by Stark et al. (2024),^51^ who analyzed methods for estimating greenhouse gas emissions from landfills. The estimated PFAS release at the lower volume of fugitive emissions was also assessed for the 25–75% quartile PFAS concentrations (Case 6). The final analysis in Table 3 (Case 7) considers the importance of the assumed ICE and flare destruction efficiency when using the lower estimate of fugitive emissions. In Case 7, because the estimated volume of LFG that is treated in a flare or ICE is static, this volume represents a higher proportion of total gas as the volume of fugitive emissions is reduced. PFAS emissions more than double when the lower volume of fugitive emissions is used with an assumed 50% PFAS destruction efficiency in flares and ICEs relative to Case 5 with 100% destruction efficiency (Table 3). Case 7 emphasizes the significance of the assumed destruction of PFAS in a flare or ICE.

This is the first study to estimate the total annual mass of volatile PFAS released in LFG from US landfills and is comparable and analogous to a previous estimate of leachate release of 600 kg annually.^13^ Although there is considerable uncertainty, PFAS emissions from US landfills are generally in the range of 100 to 2000 kg/yr for most cases. Given the range of PFAS concentrations in landfills, emissions from an individual landfill will be site-specific considering the concentration of PFAS, fugitive emissions and the destruction efficiency of the control devices. PFAS concentrations in LFG can also be influenced by the types of waste in addition to MSW that a landfill accepts. Landfills that did not accept nonhazardous industrial waste containing PFAS were shown to have lower PFAS concentrations in their leachate relative to landfills that did accept such waste.^36^

Finally, our emissions estimates assume that the landfills sampled are representative of the population of US landfills, which may not be the case. As presented in Figure 2, there is a wide distribution of SumPFAS such that a few landills with relatively high SumPAS have a strong influence on the median concentrations. Furthermore, the landfills sampled did not accept PFAS-containing nonhazardous industrial wastes that might have resulted in elevated PFAS concentrations. While site sampling was weighted toward landfills in wet regions, this is likely not problematic. Of the 30 landfills sampled, 56, 27 and 17% were in wet, moderate and arid regions, respectively. As presented in Table S14, approximately 52, 33 and 15% of LFG is generated in wet, moderate, and arid regions, respectively.

In the short term, reductions in PFAS emissions will result from increased LFG collection, assuming some destruction within the LFG treatment devices. In the long term, efforts to reduce the mass of PFAS used in consumer products that are routinely disposed of in landfills can be expected to reduce volatile PFAS release from landfills. Our findings highlight the need for research on PFAS behavior in LFG control systems. If conventional flares and ICEs do not effectively destroy PFAS, technological improvements or additional treatment might be required.

### Implications

In the global perfluoroalkyl carboxylic acids (PFCA) emissions inventory, Wang et al., 2014 ^52^ pointed out the exclusion of LFG emissions due to insufficient data. Similarly, Van Zelm et al., 2009 ^40^ analyzed the emissions of 8:2 FTOH and its subsequent transformation into PFOA due to the use of fluorotelomeracrylate polymers, which were typically incorporated into textiles and food packaging. They projected that while 8:2 FTOH and PFOA levels would decline after 2025, landfill emissions would persist since the degradation process of fluorotelomeracrylate polymers could last between 100 and 1000 years.^48,53^ Kinetic analysis of the biodegradation of fluoroacrylate polymer can also provide clues about the rate of release of PFAS through LFG. The half-life of fluoroacrylate polymer was 1200–1700 years in aerobic soil^43^ and the biodegradation rate is orders of magnitude slower in an anaerobic environment such as a landfill.^48^ Thus, a steady long-term release of PFAS from LFG can be expected. This study confirms that the release of PFAS from landfills will persist given the comparable PFAS concentrations in LFG from sites that have been closed for two to five decades (LFs 14, 22 and 23). This contrasts with the observation made in a laboratory reactor study by Ye et al.^19^ where FTOHs were released early in the decomposition cycle. It is likely that the observed release by Ye et al. can be attributed to the off-gassing of the residual FTOH monomers rather than the release of the side group from the hydrolysis of a SCFP, a process that is much slower.

The data from this study should be used to inform models to assess the expected receptor of PFAS concentrations downwind of landfills, as there is no published information on this route of exposure. Toxicokinetic modeling of the occupational hazards of ski wax technicians revealed that perfluorohexanoic acid (PFHxA), perfluoroheptanoic acid (PFHpA) and 5:3 FTCA are the primary metabolites of 6:2 FTOH after exposure by inhalation to concentrations up to 2,400 *ng*/*m* ^3^.^54,55^ Similar modeling should be performed to assess long-term volatile PFAS risks from occupational exposure, as well as exposure of communities living near landfills.

## Supplementary Material



## Notes

Detailed description of the model LFG generation and emissions from US landfills; a list of target and suspect volatile PFAS, as well as internal standards; and the results of statistical analyses (PDF)

## ∇

School of Engineering, University of North Florida, 1 UNF Drive, Jacksonville, FL 32224, USA

## References

[1] Wang Z., DeWitt J. C., Higgins C. P., Cousins I. T. (2017). A Never-Ending Story
of Per- and Polyfluoroalkyl Substances (PFASs). Environ. Sci. Technol..

[2] Evich M. G., Davis M. J. B., McCord J. P., Acrey B., Awkerman J. A., Knappe D. R. U., Lindstrom A. B., Speth T. F., Tebes-Stevens C., Strynar M. J., Wang Z., Weber E. J., Henderson W. M., Washington J. W. (2022). Per- and polyfluoroalkyl substances in the environment. Science.

[3] Buck R. C., Franklin J., Berger U., Conder J. M., Cousins I. T., de Voogt P., Jensen A. A., Kannan K., Mabury S. A., van Leeuwen S. P. (2011). Perfluoroalkyl
and polyfluoroalkyl substances in the
environment: Terminology, classification, and origins. Integrated Environmental Assessment and Management.

[4] Rewerts J. N., Morre J. T., Massey
Simonich S. L., Field J. A. (2018). In-Vial Extraction
Large Volume Gas Chromatography Mass Spectrometry for Analysis of
Volatile PFASs on Papers and Textiles. Environ.
Sci. Technol..

[5] Ritter E. E., Dickinson M. E., Harron J. P., Lunderberg D. M., DeYoung P. A., Robel A. E., Field J. A., Peaslee G. F. (2017). PIGE as
a screening tool for Per- and polyfluorinated substances in papers
and textiles. Nuclear Instruments and Methods
in Physics Research Section B: Beam Interactions with Materials and
Atoms.

[6] Robel A. E., Marshall K., Dickinson M., Lunderberg D., Butt C., Peaslee G., Stapleton H. M., Field J. A. (2017). Closing the Mass Balance on Fluorine on Papers and
Textiles. Environ. Sci. Technol..

[7] Lang J. R., Allred B. M., Peaslee G. F., Field J. A., Barlaz M. A. (2016). Release
of Per- and Polyfluoroalkyl Substances (PFASs) from Carpet and Clothing
in Model Anaerobic Landfill Reactors. Environ.
Sci. Technol..

[8] Allred B. M., Lang J. R., Barlaz M. A., Field J. A. (2015). Physical and Biological
Release of Poly- and Perfluoroalkyl Substances (PFASs) from Municipal
Solid Waste in Anaerobic Model Landfill Reactors. Environ. Sci. Technol..

[9] U.S. Environmental Protection Agency . Advancing Sustainable Materials Management: 2018 Fact Sheet Assessing Trends in Material Generation, Recycling, Composting, Combustion with Energy Recovery and Landfilling in the U.S., 2020.

[10] Barlaz M. A., Schaefer D. M., Ham R. K. (1989). Bacterial Population Development
and Chemical Characteristics of Refuse Decomposition in a Simulated
Sanitary Landfill. Appl. Environ. Microbiol..

[11] Kjeldsen P., Barlaz M. A., Rooker A. P., Baun A., Ledin A., Christensen T. H. (2002). Present
and Long-Term Composition of MSW Landfill Leachate:
A Review. Critical Reviews in Environmental
Science and Technology.

[12] Renou S., Givaudan J. G., Poulain S., Dirassouyan F., Moulin P. (2008). Landfill leachate treatment: Review and opportunity. Journal of Hazardous Materials.

[13] Lang J. R., Allred B. M., Field J. A., Levis J. W., Barlaz M. A. (2017). National
Estimate of Per- and Polyfluoroalkyl Substance (PFAS) Release to U.S.
Municipal Landfill Leachate. Environ. Sci. Technol..

[14] Huset C. A., Barlaz M. A., Barofsky D. F., Field J. A. (2011). Quantitative determination
of fluorochemicals in municipal landfill leachates. Chemosphere.

[15] Benskin J. P., Li B., Ikonomou M. G., Grace J. R., Li L. Y. (2012). Per- and Polyfluoroalkyl
Substances in Landfill Leachate: Patterns, Time Trends, and Sources. Environ. Sci. Technol..

[16] Busch J., Ahrens L., Sturm R., Ebinghaus R. (2010). Polyfluoroalkyl
compounds in landfill leachates. Environ. Pollut..

[17] Eggen T., Moeder M., Arukwe A. (2010). Municipal
landfill leachates: A significant
source for new and emerging pollutants. Science
of The Total Environment.

[18] Gallen C., Drage D., Eaglesham G., Grant S., Bowman M., Mueller J. (2017). Australia-wide assessment
of perfluoroalkyl substances
(PFASs) in landfill leachates. Journal of Hazardous
Materials.

[19] Ye Y., Titaley I. A., Kim-Fu M. L., Moll A. R., Field J. A., Barlaz M. A. (2024). Release
of Volatile Per- and Polyfluoroalkyl Substances
from Plant Fiber-Based Food Packaging and Municipal Solid Waste to
Gas under Simulated Landfill Conditions. Environ.
Sci. Technol..

[20] Lenka S. P., Kah M., Padhye L. P. (2021). A review of the occurrence, transformation, and removal
of poly- and perfluoroalkyl substances (PFAS) in wastewater treatment
plants. Water Res..

[21] Goukeh M. N., Abichou T., Tang Y. (2023). Measurement of fluorotelomer
alcohols
based on solid phase microextraction followed by gas chromatography-mass
spectrometry and its application in solid waste study. Chemosphere.

[22] Titaley I. A., De la Cruz F. B., Barlaz M. A., Field J. A. (2023). Neutral Per- and
Polyfluoroalkyl Substances in In Situ Landfill Gas by Thermal Desorption-Gas
Chromatography-Mass Spectrometry. Environmental
Science & Technology Letters.

[23] Lin A. M., Thompson J. T., Koelmel J. P., Liu Y., Bowden J. A., Townsend T. G. (2024). Landfill Gas: A Major Pathway for
Neutral Per- and
Polyfluoroalkyl Substance (PFAS) Release. Environmental
Science & Technology Letters.

[24] Tolaymat T., Robey N., Krause M., Larson J., Weitz K., Parvathikar S., Phelps L., Linak W., Burden S., Speth T., Krug J. (2023). A critical review of perfluoroalkyl
and polyfluoroalkyl substances (PFAS) landfill disposal in the United
States. Sci. Total Environ..

[26] 40 CFR Part 98 Subpart HH – Municipal Solid Waste Landfills. Mandatory Greenhouse Gas Reporting, 2009.

[27] 40 CFR Part 98 Subpart HH – Municipal Solid Waste Landfills. Revisions to the Greenhouse Gas Reporting Rule and Final Confidentiality Determinations for New or Substantially Revised Data Elements, Final Rule, 2013.

[28] Roth J., Abusallout I., Hill T., Holton C., Thapa U., Hanigan D. (2020). Release of Volatile Per- and Polyfluoroalkyl Substances
from Aqueous Film-Forming Foam. Environmental
Science & Technology Letters.

[29] Wu Y., Chang V. W.-C. (2012). Development of
analysis of volatile polyfluorinated
alkyl substances in indoor air using thermal desorption-gas chromatography-mass
spectrometry. Journal of Chromatography A.

[30] Barlaz M. A., Field J. A., S S. (2024). Characterization
and quantification
of per- and polyfluoroalkyl substances in landfill gas and estimate
of emissions from U.S. Landfills. North Carolina
State University.

[31] US Environmental Protection Agency . Inventory of U.S. Greenhouse Gas Emissions and Sinks: 1990–2020, EPA 430-P-17-001; EPA, 2022.

[32] Landfill Methane Outreach Program; U.S. Environmental Protection Agency, 2019. https://www.epa.gov/lmop (accessed 2023-9-06).

[33] U.S. EPA . AP-42 Emission factors for Municipal Solid Waste Landfills- Supplement E, ID: 142; 1998.

[34] Smallwood T. J., Robey N. M., Liu Y., Bowden J. A., Tolaymat T. M., Solo-Gabriele H. M., Townsend T. G. (2023). Per- and polyfluoroalkyl substances
(PFAS) distribution in landfill gas collection systems: leachate and
gas condensate partitioning. Journal of Hazardous
Materials.

[35] Climate at a Glance: City Time Series; NOAA National Centers for Environmental Information, 2020. https://www.ncei.noaa.gov/access/monitoring/climate-at-a-glance/city/time-series (accessed 2021-03-14).

[36] Villafuerte Patino, V. A. Does the Disposal of PFAS-containing Special Wastes Impact Leachate PFAS Concentrations? M.Sc. Thesis, North Carolina State University: Raleigh, North Carolina, 2024.

[37] Dinglasan M. J. A., Ye Y., Edwards E. A., Mabury S. A. (2004). Fluorotelomer Alcohol
Biodegradation Yields Poly- and Perfluorinated Acids. Environ. Sci. Technol..

[38] Trier X., Granby K., Christensen J. H. (2011). Polyfluorinated surfactants (PFS)
in paper and board coatings for food packaging. Environmental Science and Pollution Research.

[39] Guzman-Puyol S. (2024). Fluorinated
compounds in paper and paperboard based food packaging materials. npj Science of Food.

[40] van
Zelm R., Huijbregts M. A., Russell M. H., Jager T., van de Meent D. (2008). Modeling the environmental fate of perfluorooctanoate
and its precursors from global fluorotelomer acrylate polymer use. Environ. Toxicol. Chem..

[41] Glenn G., Shogren R., Jin X., Orts W., Hart-Cooper W., Olson L. (2021). Per- and polyfluoroalkyl substances and their alternatives in paper
food packaging. Comprehensive Reviews in Food
Science and Food Safety.

[42] FDA Announces the Voluntary Phase-Out by Industry of Certain PFAS Used in Food Packaging; US FDA, 2020; https://www.fda.gov/food/hfp-constituent-updates/fda-announces-voluntary-phase-out-industry-certain-pfas-used-food-packaging (accessed 2024-01-10).

[43] Russell M.
H., Berti W. R., Szostek B., Buck R. C. (2008). Investigation of
the Biodegradation Potential of a Fluoroacrylate Polymer Product in
Aerobic Soils. Environ. Sci. Technol..

[44] Butt C. M., Muir D. C. G., Mabury S. A. (2013). Biotransformation
pathways of fluorotelomer-based
polyfluoroalkyl substances: A review. Environ.
Toxicol. Chem..

[45] Wang N., Szostek B., Buck R. C., Folsom P. W., Sulecki L. M., Gannon J. T. (2009). 8–2 Fluorotelomer alcohol aerobic soil biodegradation:
Pathways, metabolites, and metabolite yields. Chemosphere.

[46] Zhang S., Szostek B., McCausland P. K., Wolstenholme B. W., Lu X., Wang N., Buck R. C. (2013). 6:2 and 8:2 Fluorotelomer Alcohol
Anaerobic Biotransformation in Digester Sludge from a WWTP under Methanogenic
Conditions. Environ. Sci. Technol..

[47] Hamid H., Li L. Y., Grace J. R. (2020). Formation
of perfluorocarboxylic
acids from 6:2 fluorotelomer sulfonate (6:2 FTS) in landfill leachate:
Role of microbial communities. Environ. Pollut..

[48] Washington J. W., Jenkins T. M., Rankin K., Naile J. E. (2015). Decades-Scale Degradation
of Commercial, Side-Chain, Fluorotelomer-Based Polymers in Soils and
Water. Environ. Sci. Technol..

[49] Barlaz M. A. (2006). Forest
products decomposition in municipal solid waste landfills. Waste Management.

[50] Dinglasan-Panlilio M. J., Mabury S. (2006). Significant Residual
FluorinatedAlcohols Present in
VariousFluorinated Materials. Environmental
Scince & Technology.

[51] Stark B. M., Tian K., Krause M. J. (2024). Investigation of U.S. landfill GHG
reporting program methane emission models. Waste
Management.

[52] Wang Z., Cousins I. T., Scheringer M., Buck R. C., Hungerbühler K. (2014). Global emission
inventories for C4-C14 perfluoroalkyl carboxylic acid (PFCA) homologues
from 1951 to 2030, part II: The remaining pieces of the puzzle. Environ. Int..

[53] Washington J. W., Jenkins T. M. (2015). Abiotic Hydrolysis of Fluorotelomer-Based
Polymers
as a Source of Perfluorocarboxylates at the Global Scale. Environ. Sci. Technol..

[54] Russell M. H., Himmelstein M. W., Buck R. C. (2015). Inhalation and oral toxicokinetics
of 6:2 FTOH and its metabolites in mammals. Chemosphere.

[55] Nilsson H., Kärrman A., Rotander A., van Bavel B., Lindström G., Westberg H. (2013). Biotransformation of fluorotelomer
compound to perfluorocarboxylates in humans. Environ. Int..

