# Supplementary material for: Nationwide Estimate of Volatile Per- and Polyfluoroalkyl Substance (PFAS) Emissions from U.S. Landfills via Landfill Gas
Source: Environ Sci Technol. 2025 Nov 17;59(46):24899–908. doi: 10.1021/acs.est.5c08763 (PMC12659445; doi:10.1021/acs.est.5c08763)
Supplement: Supplementary file 1 [file es5c08763_si_001.pdf]

Nationwide Estimate of Volatile Per- and Polyfluoroalkyl Substance (PFAS) Emissions from U.S. Landfills via Landfill Gas (Supporting Information)

Florentino B. De la Cruz,[*,†,‡] Ivan A. Titaley,[¶] Yixuan Wang,[†] Jennifer A. Field,[¶] and Morton A. Barlaz[†]

†*Department of Civil, Construction, and Environmental Engineering, North Carolina State University, 915 Partners Way, Raleigh, NC 27695, Raleigh, NC 27695 USA*

‡*Current Affiliation: School of Engineering, University of North Florida, 1 UNF Drive, Jacksonville, FL 32224 USA*

¶*Department of Environmental and Molecular Toxicology, Oregon State University, 1007 Agriculture and Life Sciences Building, Corvallis, Oregon 97331 USA*

E-mail: f.de.la.cruz@unf.edu

Summary

Total number of pages: S28

Total number of figures: 7

Total number of tables: 14

Contents

1 Calculation of Total PFAS Concentrations **S5**

2 Statistical Analysis of SumPFAS Upstream and Downstream of the Condensate Knockout **S5**

3 Estimate of landfill gas volume **S6**

3.1 Correction for Air Intrusion S6

3.2 Landfill Data from the Greenhouse Gas Reporting Program S7

4 Tables **S11**

5 Figures **S21**

List of Tables

S1 Characteristics of 30 study landfills across 17 states in the US. S11

S2 Target volatile PFAS. S12

S3 Suspect volatile PFAS. S12

S4 Surrogate and internal standards. S12

S5 Calibration range for PFAS analytes S13

S6 Comparison of the SumPFAS concentrations upsteream and downstream of the condensate knockout. [b] S14

S7 Secondary FTOH (sFTOH) concentration in select landfills and its percentage of the SumPFAS. S15

S8 Mean concentrations of the minor PFAS components.[a] S15

S9 Landfill status and counts by gas collection system (GCS) type for 1,124 landfills in the Greenhouse Gas Reporting Program from 2019. . . . . S16

S10 Mass of CH_4 emissions (kilotons,kt) and volume (million m^3) of LFG emissions in the U.S. by climate. CH_4 emissions were increased by 11% to account for non-reporting landfills in the GHGRP dataset. S16
S11 Range of the fraction of generated methane (modeled) that is recovered by gas collection system type. S17
S12 Summary statistics for collection efficiencies reported by landfills with a gas collection system based on the U.S. Greenhouse Gas Inventory 2021.[1] S18
S13 Modeled methane generation and measured methane recovery in million metric tons (Mt) and CH_4 collection efficiency (%) by gas collection system type and climate. S19
S14 Volumes in million m^3 (Mm^3) of LFG that is uncollected ($V_{LFG\ fugitive}$), flared ($V_{LFG\ flare}$), and converted to energy $V_{LFG\ ICE}$ using Eqs (S3) and (S4). . . S20

List of Figures

S1 QQ-Plot for SumPFAS concentration upstream (UP) and downstream (DOWN) of the condesate knockout S21
S2 Comparison of SumPFAS from open and closed landfills. S22
S3 Correlation heat map on the relationship of FTOH and sFTOH. S23
S4 Correlation heat map to evaluate the linear relationships between the PFAS and landfill characteristics S24
S5 Pairplot showing the inter-relationship between PFAS and site and environmental characteristics S25
S6 Correlation heat map of PFAS groups and environmental variables. S26

S7 Boxplot showing the assumed collection efficiencies of closed and operational landfill sites in the USGHGI. The $\times$ represents the mean of each data set, while the interior line represents the median. The boxes bound the interquartile range (IQR) (25th to 75th quartile). The whiskers extend to 1.5 times the IQR, and their ends show the highest and lowest value excluding outliers, which are represented by solid black points. S27

1 Calculation of Total PFAS Concentrations

We calculated $SumPFAS = Sum\ of\ all\ target\ PFAS$

$SumFTOH = \sum(n:2\ FTOH)$

$SumFTO = \sum(n:2\ FTO)$

$SumFOSA = \sum(N-MeFOSA,\ N-EtFOSA)$

$SumFOSE = \sum(N-MeFOSE,\ N-EtFOSE)$

$SumFTAc = \sum(n:2\ FTAc)$

$SumFTMAc = \sum(n:2\ FTMAc)$

$SumFTI = \sum(n:2\ FTI)$

where n is the number of fully fluorinated carbon in the molecule; A complete list of analytes, internal and surrogate standards is presented in Tables S2 to S4 of the SI.

2 Statistical Analysis of SumPFAS Upstream and Downstream of the Condensate Knockout

We hypothesized that the elimination of moisture in the condensate knockout of the main header would result in a reduction in PFAS because some PFAS would condense and some are likely bound to the particulate phase. To test this hypothesis, we performed the Kruskal-Wallis and Mann-Whitney U statistic test to compare PFAS in samples collected upstream (UP) and downstream (DOWN) of the condensate knockout system. These statistical tests are more appropriate due to the nonnormality of the dataset. The results of the Kruskal-Wallis test are as follows: Kruskal-Wallis statistic = 1.94; p-value = 0.164. The Kruskal-Wallis statistic value indicates the extent to which the sumPFAS distributions between the UP and DOWN samples differ. A higher value would suggest a greater difference between the groups. The p-value of approximately 0.164 is greater than the significance level $\alpha = 0.05$ (or 5%). This result indicates that the distribution of SumPFAS upstream and downstream

of condensate knockout is not significantly different. This conclusion is supported by the Mann-Whitney U statistic of 12,704 and the p-value = 0.164.

3 Estimate of landfill gas volume

The mass of PFAS released in US landfill gas (LFG) was estimated from the model given in Equation 1 of the manuscript. A national inventory of LFG generation, collection, treatment, and fugitive emissions was developed using data from the US EPA Greenhouse Gas Inventory (USGHGI)[1] and the US EPA Landfill Methane Outreach Program (LMOP) database.[2] Measured PFAS concentrations were applied to the developed LFG inventory to estimate PFAS emissions from US landfills.

3.1 Correction for Air Intrusion

Air intrusion is common in gas collection systems, resulting in the detection of O_2 in LFG. To match the inherent anaerobic landfill assumption (50/50, CH_4 /CO_2) associated with the estimate of national landfill gas emissions, the SumPFAS quantile ranges were derived from the concentration distributions for different precipitation regions after correction for air intrusion.[3]

$$C_{\text{PFAS, cor}} = C_{\text{PFAS}} \times \frac{100}{C_{\text{CH}_4} + C_{\text{CO}_2}} \tag{S1}$$

Where:

$C_{\text{PFAS, cor}}$ = air-corrected PFAS concentration (ng/m^3)

C_{PFAS} = measured PFAS concentration (ng/m^3)

C_{CH_4} = LFG CH_4 concentration

C_{CO_2} = LFG CO_2 concentration

3.2 Landfill Data from the Greenhouse Gas Reporting Program

The USGHGI estimates landfill methane emissions based on self-reported facility data from the Greenhouse Gas Reporting Program (GHGRP),[4,5] which requires MSW landfills that emit >25,000 metric tons of CO_2 equivalents to report operating data (e.g., landfill status, gas recovery status and measurement methods, modeled methane generation, measured methane recovery). A total of 1,124 landfills were included in the 2019 data. The USGHGI includes two approaches to estimate the LFG generation. The first is based on the Intergovernmental Panel on Climate Change (IPCC) first-order degradation (FOD) model,[6] which is similar to the US EPA Landfill Gas Emissions Model (LandGEM). Modeled methane generation is based on waste disposal data available only to the landfill owner and, therefore, likely more accurate than alternatives to independently model methane generation. The second method used in the USGHGI to estimate LFG generation uses the measured LFG recovery and an estimate of the fraction of generated LFG that is collected. Both methods are described here.

Although the GHGRP database identifies whether a landfill has a gas collection system (GCS), it does not indicate whether the collected LFG is flared or converted to energy. This information is desirable so that models of PFAS emissions can incorporate distinct destruction efficiencies for different types of gas treatment systems (e.g., flare, internal combustion engine [ICE]). Fortunately, the LMOP database (September 2021)[2] includes data on the types and status of LFG energy projects for municipal solid waste (MSW) landfills and was therefore integrated with GHGRP data to categorize landfills with GCS into flare and energy recovery scenarios (Table S9). The LMOP database includes 1,319 landfills that have unique GHGRP IDs and report data to the GHGRP. Only two of the 1,124 landfills that are included in the GHGRP database are not in the LMOP database. These two landfills passively vent their LFG and are therefore not candidates for beneficial CH_4 recovery. Thus, the LMOP database includes all LFG energy projects in the GHGRP.

For landfills without a GCS in place, CH_4 emissions reported in the USGHGI were estimated based on the modeled CH_4 generation ($G_{\mathrm{CH_4}}$) using the IPCC FOD model and assumed cover type-dependent oxidation efficiencies (OX) (Eq. S2).[6] For landfills with a GCS, the USGHGI applies two approaches to self-reported GHGRP data to estimate CH_4 emissions. The first approach calculates CH_4 emissions based on modeled CH_4 generation using the IPCC's FOD model, which is then reduced by the measured recovery (R), the assumed oxidation of uncollected methane, the CH_4 destruction efficiency in a gas treatment device (flare or ICE) (DE) and the fraction of hours (f_{Dest}) that the treatment device is operating (Eq. S3). The second approach calculates CH_4 emissions from the measured recovered CH_4, an assumed collection efficiency (CE), the fraction of hours that the recovery system is operating (f_{Rec}), and oxidation (Eq. S4). The USGHGI methodology suggests applying a scaling factor of 11% to the calculated CH_4 emissions of the GHGRP data for 2005 - 2019 to account for landfills that are not subject to the GHGRP. The EPA estimated this scale-up factor based on the amount of waste in place in landfills in the US. CH_4 emissions, as reported by the EPA, divided by climatic region, are presented in Table S10. Specific precipitation information for each GHGRP landfill was incorporated using the geographic location (latitude and longitude) in the LMOP (2021) database[2] and the average annual precipitation of the local area.[7] This allocation allows us to assign different concentrations of PFAS to landfills in arid, moderate, and wet regions as appropriate.

$$\mathrm{CH_4}Emissions_{NoGCS} = G_{\mathrm{CH_4}} \times (1 - OX) \tag{S2}$$

$$\mathrm{CH_4}Emissions_{GCS} = (G_{\mathrm{CH_4}} - R) \times (1 - OX) + R(1 - DE \times f_{Dest}) \tag{S3}$$

$$\mathrm{CH_4}Emissions_{GCS} = \left(\frac{R}{CE \times f_{Rec}} - R\right) \times (1 - OX) + R(1 - DE \times f_{Dest}) \tag{S4}$$

The volume of fugitive LFG $V_{fugitive\,LFG,i})$ is the total volume of LFG that escapes through the soil cover. It was estimated based on the volume estimates of CH_4 generation ($V_{CH_4\,generated,i}$) and recovery in a flare or engine ($V_{CH_4\,flared,i}$), ($V_{CH_4\,ICE}, i$), assuming that the LFG is 50% CH_4 (Eq S5). $V_{CH_4\,generated,i}$ includes the gas generated from landfills with and without a GCS. Although some CH_4 is converted to CO_2 in the soil, this does not change the volume of the gas emitted.

$$V_{fugitive\,LFG,i} = \frac{V_{CH_4\,generated,i} - V_{CH_4\,flared,i} - V_{CH_4\,ICE,i}}{0.5} \tag{S5}$$

To estimate the volumes of CH_4 generated ($V_{CH_4\,generated,i}$), flared ($V_{CH_4\,flared,i}$), and converted to energy ($V_{CH_4\,ICE,i}$), two methods were used corresponding to Eqs (S3) and (S4). The first approach applies the modeled methane generation (G_{CH_4}) reported using the FOD model and the measured recovery CH_4 (R) in Eq. (S3). Of 830 landfills that recovered CH_4 in a flare or an engine, six did not report recovered CH_4 (R in Eq. S3) and 68 reported recovered CH_4 above the modeled generation (Table S13), so adjustments were needed for these 74 landfills. As measured CH_4 recovery data is assumed to be more accurate than modeled generation, CH_4 recovery was kept constant for landfills that reported measured recovery. For the six landfills with missing recovery data CH_4, recovery was estimated based on the emissions reported using Eq. (S4), the assumed oxidation and collection efficiency, and an assumed flare or engine destruction efficiency of 100%. To estimate the LFG generation for the 68 landfills in which R exceeded G_{CH_4}, lower and upper estimates of the fraction of CH_4 recovered from the modeled generation were developed as presented in Table S11. An upper estimate was developed assuming that the ratio of recovered to generated CH_4 was 95% and 90% for closed and open landfills, respectively, based on the reported data (Table S12 and Figure S7).[1] The lower estimate of this ratio was determined by replacing the mean values grouped by GCS type in landfills with ratios $< 95\%$ and $< 90\%$ for each closed and open landfill with the collected efficiency reported $> 95\%$ and $> 90\%$, respectively. The mean values of the GCS group were used to differentiate the ratios between landfills with flares

and engines because they showed different ratios of gas collected to gas generated (Table S13). Table S13 shows the mass estimates of CH_4 generated, flared and converted to energy, which were converted to respective volumes ($V_{\mathrm{CH_4}\,generated,i}$, $V_{\mathrm{CH_4}\,flared,i}$, and $V_{\mathrm{CH_4}\,ICE,i}$) using a density of CH_4 of 0.657 kgm^{-3} at $25^{\circ}\,C$. These CH_4 volumes were doubled and include the 11% scale-up factor to the volumes of LFG generated, flared and converted to energy (Table S14).

In the second approach to estimate $V_{\mathrm{CH_4}\,generated,i}$, $V_{\mathrm{CH_4}\,flared,i}$ and $V_{\mathrm{CH_4}\,forenergy,i}$, the measured recovery and assumed collection efficiencies CH_4 directly reported by the facilities were used to estimate the CH_4 generated $\left(\frac{R}{CE}\right)$ as part of Eq. (S4). A closed landfill site with a flare did not report the assumed collection efficiency, and the mean collection efficiency of 86% was assumed from the remaining closed landfills with flare systems. For the six landfills with missing data on recovered CH_4, recovery was estimated based on modeled CH_4 generation and self-reported collection efficiency. The estimated ranges for CH_4 generation and the fraction of CH_4 recovery corresponding to Eq (S3) and Eq (S4) are summarized by type of GCS and climate in Table S14.

The volumes of LFG that are generated, uncollected ($V_{fugitive\,LFG,i}$), flared ($V_{LFG\,flared,i}$), and converted to energy ($V_{ICE,i}$) for each climate region using two methods are presented in Table S14. Estimates of LFG emissions using Eq. (S3) are 2 to 2.4 times higher than the estimates of emissions using Eq. S4. A possible explanation for this is that the LFG generation is estimated using Eq. (S4) applied a site-reported collection efficiency (CE) that could be biased high. Higher collection efficiencies (67%) were obtained using Eq (S4) than those with Eq (S3) (52%) (Table S13). Another possible explanation is that G is overestimated in Eq (S3). Therefore, the corresponding fugitive gas emissions and corresponding PFAS emissions will vary by a factor of > 2 based on uncertainty in estimates of LFG emissions alone.

4 Tables

Table S1: Characteristics of 30 study landfills across 17 states in the US.

**GHGRP Class**[a]	**Alias**	**Status**	**Age (yr)**[b]	**ppt (mm)**[c]
Wet	LF1	Open	49	1228
Wet	LF11	Open	69	1064
Wet	LF12	Open	30	1341
Wet	LF13	Open	16	1315
Wet	LF15	Open	46	1228
Wet	LF16	Open	43	1302
Wet	LF18	Open	41	1413
Wet	LF19	Open	42	1463
Wet	LF2	Open	21	1153
Wet	LF21	Open	70	1648
Wet	LF23	Closed	35	1183
Wet	LF24	Open	43	1590
Wet	LF26	Open	13	1176
Wet	LF28	Open	21	1369
Wet	LF29	Open	48	1079
Wet	LF30	Open	25	1249
Wet	LF4	Open	44	1262
Moderate	LF10	Open	43	1006
Moderate	LF14	Closed	76	1001
Moderate	LF17	Open	40	837
Moderate	LF20	Open	45	1004
Moderate	LF22	Closed	44	979
Moderate	LF25	Open	39	887
Moderate	LF6	Open	48	923
Moderate	LF8	Open	44	812
Arid	LF27	Open	44	391
Arid	LF3	Open	47	327
Arid	LF5	Open	44	290
Arid	LF7	Open	51	226
Arid	LF9	Open	31	443

[a] GHGRP precipitation classification based on ref.[4,5]
[b] Landfill age calculated as the difference between the sampling date and the date the landfill opened.
[c] NOAA Annual normal precipitation at the closest weather station.[7]

Table S2: Target volatile PFAS.

Analyte	Abbreviation	Vendor[a]	MW (a.m.u.)	CAS no.	Formula	Quantifier ion (m/z)	Qualifier ion (m/z)	Surrogate standard
4:2 fluorotelomer alcohol	4:2 FTOH	Wellington	264	2043-47-2	$C_6H_5F_9O$	265	227	MFBET
6:2 fluorotelomer alcohol	6:2 FTOH	Wellington	364	647-42-7	$C_8H_5F_{13}O$	365	327	MFHET
8:2 fluorotelomer alcohol	8:2 FTOH	Wellington	464	678-39-7	$C_{10}H_5F_{17}O$	465	427	M2FOET
10:2 fluorotelomer alcohol	10:2 FTOH	Wellington	564	865-86-1	$C_{12}H_5F_{21}O$	565	527	MFDET
12:2 fluorotelomer alcohol	12:2 FTOH	SynQuest	664	39239-77-5	$C_{14}H_5F_{25}O$	665	627	MFDET
N-methyl perfluorooctane sulfonamide	MeFOSA	Wellington	513	31506-32-8	$C_9H_4NO_2SF_{17}$	514	-	d3-N-MeFOSA-M
N-ethyl perfluorooctane sulfonamide	EtFOSA	Wellington	527	4151-50-2	$C_{10}H_6NO_2SF_{17}$	528	-	d5-N-EtFOSA-M
N-methyl perfluorooctane sulfonamidoethanol	MeFOSE	Wellington	557	24448-09-7	$C_{11}H_8NO_3SF_{17}$	540	558	d7-N-MeFOSE-M
N-ethyl perfluorooctane sulfonamidoethanol	EtFOSE	Wellington	571	1691-99-2	$C_{12}H_{10}NO_3SF_{17}$	554	572	d9-N-EtFOSE-M
6:2 fluorotelomer acrylate	6:2 FTAc	SynQuest	418	17527-29-6	$C_{11}H_7F_{13}O_2$	419	-	d5-6:2 FTMAc
8:2 fluorotelomer acrylate	8:2 FTAc	Wellington	518	27905-45-9	$C_{13}H_7F_{17}O_2$	519	-	d5-6:2 FTMAc
10:2 fluorotelomer acrylate	10:2 FTAc	Wellington	618	17741-60-5	$C_{15}H_7F_{21}O_2$	619	-	d5-6:2 FTMAc
6:2 fluorotelomer methylacrylate	6:2 FTMAc	SynQuest	432	2144-53-8	$C_{12}H_9F_{13}O_2$	433	461	d5-6:2 FTMAc
8:2 fluorotelomer methylacrylate	8:2 FTMAc	SynQuest	532	1996-88-9	$C_{14}H_9F_{17}O_2$	533	561	d5-6:2 FTMAc
6:2 fluorotelomer olefin	6:2 FTO	SynQuest	346	25291-17-2	$C_8H_3F_{13}$	327	-	d5-6:2 FTMAc
8:2 fluorotelomer olefin	8:2 FTO	Matrix	446	21652-58-4	$C_{10}H_3F_{17}$	427	-	d5-6:2 FTMAc
10:2 fluorotelomer olefin	10:2 FTO	Matrix	546	30389-25-4	$C_{12}H_3F_{21}$	527	-	d5-6:2 FTMAc
12:2 fluorotelomer olefin	12:2 FTO	SynQuest	646	67103-05-3	$C_{14}H_3F_{25}$	627	-	d5-6:2 FTMAc
Perfluorohexyl iodide	PFHxI	SynQuest	446	355-43-1	$C_6F_{13}I$	319	427	d5-6:2 FTMAc
Perfluorooctyl iodide	PFOI	SynQuest	546	507-63-1	$C_8F_{17}I$	419	527	d5-6:2 FTMAc
Perfluorodecyl iodide	PFDI	SynQuest	646	423-62-1	$C_{10}F_{21}I$	519	627	d5-6:2 FTMAc
4:2 fluorotelomer iodide	4:2 FTI	SynQuest	374	2043-55-2	$C_6H_4F_9I$	355	403	d5-6:2 FTMAc
6:2 fluorotelomer iodide	6:2 FTI	SynQuest	474	2043-57-4	$C_8H_4F_{13}I$	455	503	d5-6:2 FTMAc
8:2 fluorotelomer iodide	8:2 FTI	SynQuest	574	2043-53-0	$C_{10}H_4F_{17}I$	555	603	d5-6:2 FTMAc
10:2 fluorotelomer iodide	10:2 FTI	SynQuest	674	2043-54-1	$C_{12}H_4F_{21}I$	655	703	d5-6:2 FTMAc

[a] Wellington: Wellington Laboratories (Guelph, ON), SynQuest: SynQuest Laboratories (Alachua, FL), Matrix: Matrix Scientific (Columbia, SC).

Table S3: Suspect volatile PFAS.

Analyte	Abbreviation	MW(a.m.u.)	CAS no.	Formula	Quantifier ion (m/z)	Qualifier ion (m/z)	Calibration for semi quantification
14:2 fluorotelomer alcohol	14:2 FTOH	764	60699-51-6	$C_{14}H_2F_{27}O$	765	727	10:2 FTOH
N-methyl perfluorobutane sulfonamidoethanol	MeFBSA	313	68298-12-4	$C_5H_{10}NO_2SF_3$	314	-	MeFOSA
N-methyl perfluorohexane sulfonamidoethanol	MeFHxSA	413	68259-15-4	$C_7H_{10}NO_2SF_{13}$	414	-	MeFOSA
N-methyl perfluoropropane sulfonamidoethanol	MeFPtSE	307	Not found	$C_4H_{10}NO_2SF_7$	290	308	MeFOSE
N-methyl perfluorobutane sulfonamidoethanol	MeFBSE	357	34454-97-2	$C_5H_{10}NO_2SF_9$	340	358	MeFOSE
N-methyl perfluoropentane sulfonamidoethanol	MeFPtSE	407	68555-74-8	$C_6H_{10}NO_2SF_{11}$	390	408	MeFOSE
N-methyl perfluorohexane sulfonamidoethanol	MeFHxSE	457	68555-75-9	$C_7H_{10}NO_2SF_{13}$	440	458	MeFOSE
N-methyl perfluoroheptane sulfonamidoethanol	MeFHpSE	507	68555-76-0	$C_8H_{10}NO_2SF_{15}$	490	508	MeFOSE
N-ethyl perfluorohexane sulfonamidoethanol	EtFHxSE	271	Not found	$C_7H_{12}NO_2SF_{17}$	254	272	EtFOSE
N-ethyl perfluoropropane sulfonamidoethanol	EtFPtSE	321	Not found	$C_4H_{12}NO_2SF_7$	304	322	EtFOSE
N-ethyl perfluorobutane sulfonamidoethanol	EtFBSE	371	34454-97-1	$C_5H_{12}NO_2SF_9$	354	372	EtFOSE
N-ethyl perfluoropentane sulfonamidoethanol	EtFPtSE	421	68555-72-6	$C_6H_{12}NO_2SF_{11}$	404	422	EtFOSE
N-ethyl perfluorohexane sulfonamidoethanol	EtFHxSE	471	34455-03-3	$C_7H_{12}NO_2SF_{13}$	454	472	EtFOSE
N-ethyl perfluoroheptane sulfonamidoethanol	EtFHpSE	521	68555-73-7	$C_8H_{12}NO_2SF_{17}$	504	522	EtFOSE

Table S4: Surrogate and internal standards.

Surrogate Standard	Abbreviation	Vendor	Molecular Weight (a.m.u)	Formula	Quantifier ion (m/z)	Qualifier ion (m/z)	Internal Standard
2-perfluorobutyl-[1,1,2,2-$^2H_4$]-ethanol	MFBET	Wellington	268	$C_6D_4HOF_9$	269	-	7:1 FTAc
2-perfluorohexyl-[1,1,2,2-$^{13}C_2$]-ethanol	MFHET	Wellington	368	$^{13}C^{12}_2C_4D_2H_2OF_{13}$	369	-	7:1 FTAc
2-perfluorooctyl-[1,1,2H_2]-[1,2-$^{13}C_2$]-ethanol	M2FOET	Wellington	463	$^{13}C^{12}_2C_6D_2H_3OF_{17}$	467	-	7:1 FTAc
2-perfluorodecyl-[1,1-$^2H_2$]-[1,2-$^{13}C_2$]-ethanol	MFDET	Wellington	568	$^{13}C^{12}_2C_8D_2H_3OF_{21}$	568	-	7:1 FTAc
N-methyl-d_3-perfluoro-1-octanesulfonamide	d_3-N-MeFOSA-M	Wellington	516	$C_9D_3HNO_2SF_{17}$	514	-	7:1 FTAc
N-ethyl-d_5-perfluoro-1-octanesulfonamide	d_5-N-EtFOSA-M	Wellington	532	$C_{10}D_5HNO_2SF_{17}$	523	-	7:1 FTAc
2-(N-methyl-d_3-perfluoro-1-octanesulfonamido)ethanol-d_3	d_7-N-MeFOSE-M	Wellington	565	$C_{11}D_7HNO_3SF_{17}$	565	-	7:1 FTAc
2-(N-ethyl-d_5-perfluoro-1-octanesulfonamido)ethanol-d_3	d_8-N-EtFOSE-M	Wellington	580	$C_{12}D_8HNO_3SF_{17}$	581	-	7:1 FTAc
1H,1H,2H,2H, Perfluoro-n-octyl methacrylate-d_3	d_3-FOMA	Sapphire	437	$C_{12}D_3H_9F_{13}O_2$	438	-	7:1 FTAc
1H,1H, Perfluorooctyl acrylate [a]	-	SynQuest	414	$C_{11}H_7F_{13}O_2$	435	-	7:1 FTAc

[a] Internal Standard

Table S5: Calibration range for PFAS analytes

**Analyte**	**Calibration curve range (pg on TD tube)**	**Fit**
4:2 FTOH	10 – 5000	Quadratic
6:2 FTOH	50 – 5000; 5000 – 250000	Quadratic; Linear
8:2 FTOH	10 – 5000	Quadratic
10:2 FTOH	10 – 5000	Quadratic
12:2 FTOH	5 – 5000	Linear
N-MeFOSA	25 – 2000	Quadratic
N-EtFOSA	25 – 2000	Linear
N-MeFOSE	50 – 5000	Quadratic
N-EtFOSE	50 – 5000	Quadratic
4:2 FTAC	5 – 500	Quadratic
6:2 FTAC	50 – 2000	Quadratic
8:2 FTAC	25 – 2000	Quadratic
10:2 FTAC	5 – 500	Quadratic
12:2 FTAC	25 – 5000	Linear
4:2 FTO	5 – 500	Quadratic
6:2 FTO	5 – 500	Quadratic
8:2 FTO	10 – 500	Quadratic
10:2 FTO	5 – 500	Quadratic
PFOI	25 – 2000	Quadratic
PFPI	50 – 2000	Quadratic
4:2 FTI	1 – 250	Quadratic
6:2 FTI	1 – 250	Quadratic
8:2 FTI	1 – 250	Quadratic
10:2 FTI	1 – 250	Quadratic

Table S6: Comparison of the SumPFAS concentrations upsteream and downstream of the condensate knockout. [b]

**Alias**	**Sample**	**SumPFAS** (ng/m^3)
LF1	UP	1.74×10^4
LF1	DOWN	9.24×10^3
LF2	UP	1.88×10^4
LF2	DOWN	1.89×10^4
LF3	UP	4.68×10^3
LF3	DOWN	4.28×10^3
LF4	UP	1.53×10^3
LF4	DOWN	6.29×10^3
LF5	UP	7.19×10^4
LF5	DOWN	4.58×10^4
LF6	UP	9.47×10^4
LF6	DOWN	8.81×10^4
LF7	UP	8.97×10^4
LF7	DOWN	9.41×10^4
LF8	UP	9.09×10^4
LF8	DOWN	8.48×10^4
LF9	UP	1.76×10^5
LF9	DOWN	1.67×10^5
LF10	UP	3.30×10^4
LF10	DOWN	2.48×10^4
LF11	UP	3.26×10^4
LF11	DOWN	6.71×10^4
LF12	UP	3.07×10^4
LF12	DOWN	3.47×10^3
LF13	UP	1.64×10^3
LF13	DOWN	1.68×10^4
LF14	UP	4.12×10^3
LF14	DOWN	1.77×10^3
LF15	UP	2.30×10^2
LF15	DOWN	1.60×10^5
LF16	UP	3.16×10^3
LF16	DOWN	1.71×10^3
LF17	UP	2.91×10^4
LF17	DOWN	1.26×10^4
LF18	UP	2.91×10^3
LF18	DOWN	2.47×10^4
LF19	UP	5.34×10^4
LF19	DOWN	9.52×10^4
LF20	UP	1.82×10^5
LF20	DOWN	1.94×10^5
LF21	UP	5.60×10^3
LF21	DOWN	7.94×10^3
LF22	UP	1.86×10^3
LF22	DOWN	2.12×10^3
LF23	UP	4.42×10^3
LF23	DOWN	3.74×10^4
LF24	UP	2.42×10^4
LF24	DOWN	3.61×10^4
LF25	UP	1.01×10^5
LF25	DOWN	9.07×10^4
LF26	UP	1.34×10^4
LF26	DOWN	7.91×10^3
LF27	UP	2.39×10^3
LF27	DOWN	3.00×10^3
LF28	UP	1.42×10^4
LF28	DOWN	1.37×10^4
LF29[b]	UP	3.55×10^4
LF30	UP	3.16×10^4
LF30	DOWN	2.22×10^4

[a] UP - Upstream of the condensate knockout, DOWN - downstream of the condensate knockout

[b] LF29 DOWN samples were not successfully analyzed.

Table S7: Secondary FTOH (sFTOH) concentration in select landfills and its percentage of the SumPFAS.

Alias	3:2 sFTOH (ng/m^3)	5:2 sFTOH (ng/m^3)	7:2 sFTOH (ng/m^3)	SumPFAS (ng/m^3)	Sum-sFTOH (ng/m^3)	% Sum-sFTOH
LF1	0.0×10^0	7.8×10^1	5.1×10^3	4.2×10^4	5.2×10^3	10.9
LF2	0.0×10^0	1.2×10^2	2.6×10^2	6.6×10^4	3.8×10^2	0.6
LF3	0.0×10^0	8.7×10^1	0.0×10^0	4.5×10^3	8.7×10^1	1.9
LF4	0.0×10^0	8.9×10^1	1.5×10^2	2.3×10^3	2.4×10^2	9.3
LF5	0.0×10^0	0.0×10^0	0.0×10^0	1.2×10^5	0.0×10^0	0.0
LF8	0.0×10^0	2.3×10^2	1.4×10^2	7.4×10^4	3.8×10^2	0.5
LF9	1.4×10^2	2.5×10^2	0.0×10^0	1.3×10^5	3.9×10^2	0.3
LF10	0.0×10^0	2.6×10^1	1.2×10^2	2.2×10^3	1.4×10^2	6.2
LF11	0.0×10^0	6.2×10^1	1.5×10^2	4.5×10^3	2.2×10^2	4.7
LF12	1.5×10^1	8.3×10^1	7.6×10^0	2.7×10^4	1.0×10^2	0.4
LF14	0.0×10^0	0.0×10^0	0.0×10^0	7.4×10^2	0.0×10^0	0.0
LF17	0.0×10^0	7.4×10^1	1.6×10^1	2.7×10^3	9.0×10^1	3.2
LF19	2.0×10^0	6.4×10^2	3.7×10^2	1.2×10^5	1.0×10^3	0.8
LF21	0.0×10^0	1.0×10^2	5.7×10^1	6.8×10^3	1.6×10^2	2.3
LF22	0.0×10^0	0.0×10^0	2.0×10^1	2.1×10^3	2.0×10^1	1.0
LF24	1.3×10^2	1.1×10^2	0.0×10^0	5.1×10^4	2.5×10^2	0.5
LF25	0.0×10^0	4.8×10^2	1.7×10^2	1.3×10^5	6.5×10^2	0.5
LF26	0.0×10^0	6.4×10^1	0.0×10^0	5.4×10^3	6.4×10^1	1.2
LF27	0.0×10^0	3.5×10^1	4.6×10^1	2.7×10^3	8.1×10^1	2.9
LF29	0.0×10^0	3.6×10^2	2.8×10^2	3.8×10^4	6.4×10^2	1.7
LF30	0.0×10^0	7.7×10^1	1.3×10^2	2.8×10^4	2.0×10^2	0.7

Table S8: Mean concentrations of the minor PFAS components.[a]

GHGRP Class	Alias	SumFTO (ng/m^3) [b]	SumFTAc (ng/m^3)	SumFTMAc(ng/m^3)	SumFOSA (ng/m^3)	SumFOSE (ng/m^3)	SumFTI (ng/m^3)
Wet	LF1	3400 (3676)	2 (4)	1 (3)	7 (23)	69 (164)	66 (128)
Wet	LF11	59 (10)	0 (0)	0 (0)	0 (0)	0 (0)	0 (0)
Wet	LF12	177 (58)	0 (0)	0 (0)	17 (29)	12 (24)	0 (0)
Wet	LF13	619 (283)	0 (0)	0 (1)	0 (0)	46 (52)	31 (55)
Wet	LF15	161 (237)	0 (0)	3 (7)	0 (0)	0 (0)	0 (0)
Wet	LF16	889 (796)	24 (37)	5 (11)	0 (0)	0 (0)	17 (36)
Wet	LF18	3052 (5785)	28 (52)	0 (0)	8 (28)	32 (45)	35 (64)
Wet	LF19	881 (873)	3 (9)	0 (0)	0 (0)	12 (37)	0 (0)
Wet	LF2	736 (544)	0 (0)	0 (0)	0 (0)	0 (0)	0 (0)
Wet	LF21	130 (6)	0 (0)	0 (0)	0 (0)	35 (41)	0 (0)
Wet	LF23	1921 (2705)	22 (51)	4 (11)	10 (34)	6 (21)	0 (0)
Wet	LF24	220 (77)	0 (0)	0 (0)	0 (0)	187 (622)	0 (0)
Wet	LF26	980 (822)	3 (13)	0 (0)	1 (5)	2 (11)	0 (0)
Wet	LF28	501 (529)	0 (0)	0 (0)	7 (17)	56 (101)	17 (26)
Wet	LF29	629 (284)	0 (0)	0 (0)	0 (0)	0 (0)	0 (0)
Wet	LF30	348 (23)	0 (0)	0 (0)	0 (0)	0 (0)	0 (0)
Wet	LF4	223 (83)	28 (51)	0 (0)	11 (21)	0 (0)	0 (0)
Moderate	LF10	1362 (1115)	8 (19)	9 (19)	10 (27)	0 (0)	5 (16)
Moderate	LF14	1478 (1684)	1 (2)	0 (1)	0 (0)	22 (85)	93 (139)
Moderate	LF17	186 (148)	0 (0)	0 (0)	0 (0)	0 (0)	0 (0)
Moderate	LF20	291 (188)	4 (13)	3 (12)	19 (49)	0 (0)	0 (0)
Moderate	LF22	0 (0)	0 (0)	0 (0)	0 (0)	0 (0)	0 (0)
Moderate	LF25	757 (848)	0 (0)	1 (3)	0 (0)	0 (0)	0 (0)
Moderate	LF6	85 (27)	1 (3)	0 (0)	0 (0)	0 (0)	0 (0)
Moderate	LF8	180 (104)	0 (0)	0 (0)	0 (0)	0 (0)	0 (0)
Arid	LF27	603 (437)	0 (0)	0 (0)	0 (0)	0 (0)	0 (0)
Arid	LF3	151 (15)	0 (0)	0 (0)	0 (0)	0 (0)	0 (0)
Arid	LF5	12651 (10453)	21 (47)	4 (13)	0 (0)	0 (0)	7 (24)
Arid	LF7	689 (948)	4 (14)	1 (5)	4 (12)	0 (0)	6 (19)
Arid	LF9	418 (150)	0 (0)	0 (0)	0 (0)	0 (0)	0 (0)

[a] Standard deviations (SD) presented parenthetically.
[b] Of the minor components analyzed FTO was consistently detected in all landfills.

Table S9: Landfill status and counts by gas collection system (GCS) type for 1,124 landfills in the Greenhouse Gas Reporting Program from 2019.

**Landfill Status**	**Total Count**	**Count by GCS Type**		
		**No Active GCS**	**Flare**	**ICE**
Operational	941	254	398	289
Closed	183	40	130	13
**Total**	1124	294	528	302

Table S10: Mass of CH_4 emissions (kilotons,kt) and volume (million m^3) of LFG emissions in the U.S. by climate. CH_4 emissions were increased by 11% to account for non-reporting landfills in the GHGRP dataset.

**Climate**	**Annual precipitation (cm)**	**Waste decay rate** $(yr^{-}1)^{1}$	**kt CH_4 emissions Eq (S3)**[a]	**kt CH_4 emissions Eq (S4)**[a]	**million m^3 LFG emissions Eq (S3)** [b]	**million m^3 LFG emissions Eq (S4)**[b]
**Arid**	<51	0.02	866	491	2,636	1,495
**Moderate**	51–102	0.038	1,331	1,064	4,052	3,239
**Wet**	>102	0.057	3,236	1,799	9,851	5,476
**Total**			**5,433**	**3,354**	**16,539**	**10,210**

[a] One of 1,124 landfills did not report emissions using Eq (S3), and 4 of 1,124 landfills did not report emissions using Eq (S4). The median percent difference (54%) of emissions for individual landfills between Eq (S3) and Eq (S4) was used to scale the emissions from Eq (S3) or Eq (S4) to fill in the missing estimates.

[b] Volume of LFG emissions were calculated assuming that LFG is 50% CH_4 and a CH4 density of 0.657 kg m-3 at 25 ^{o}C. The volume is reduced by oxidized CH_4.

Table S11: Range of the fraction of generated methane (modeled) that is recovered by gas collection system type.

Range of Percentage of the Modeled CH_4 Generation this is Recovered	Range Grouped Count	Count by GCS Type			Assumptions for Lower and Upper Ratio Estimates of CH_4 Recovered over Modeled Generation		Estimates Adjusted by Lower and Upper % CH_4 Recovered	
		No Active GCS	Flare	Energy Recovery	Lower	Upper	Measured Recovery	Annual Modeled CH_4 Generation
**183 Closed Landfills**								
**0%**	40	40	-	-	NA	NA	NA	Reported G_{CH_4}
**(0%, 95%]**	132	-	121	11	No change $(\frac{R}{G_{CH_4}})$	No change $(\frac{R}{G_{CH_4}})$	Reported R	Reported G_{CH_4}
**(95%, 100%]**	2	-	1	1	**Mean**[b]	95%	Reported R	Lower: $R/0.95$; Upper: $R/mean\%$ *recovered*
**>100%**	8	-	**7**[a]	1	**Mean**[b]	95%	Reported R	Lower: $R/0.95$; Upper: $R/mean\%$ *recovered*
**NA**	1	-	1	0	Estimated $(\frac{R}{G_{CH_4}})$	No change $(\frac{R}{G_{CH_4}})$	Estimated R using emissions reported in Eq (S3) $R = G_{CH_4} - \frac{CH_4\ Emissions_{GCS}}{(1-OX)}$	Reported G_{CH4}
**Sub-total**	**183**	**40**	**130**	**13**				
**941 Open Landfills**								
**0%**	254	254	-	-	NA	NA	NA	Reported G_{CH_4}
**(0%, 90%]**	588	-	346	242	No change $(\frac{R}{G_{CH_4}})$	No change $(\frac{R}{G_{CH_4}})$	Reported R	Reported G_{CH4}
**(90%, 100%]**	34	-	17	17	**Mean**[c]	90%	Reported R	Lower: $R/0.95$; Upper: $R/mean\%$ *recovered*
**>100%**	60	-	**33**[a]	27	**Mean**[c]	90%	Reported R	Lower: $R/0.95$; Upper: $R/mean\%$ *recovered*
**NA**	5	-	2	3	Estimated (R/G_{CH_4})	Estimated (R/G_{CH_4})	Estimated R using emissions reported in Eq (S3) $R = G_{CH_4} - \frac{CH_4\ Emissions_{GCS}}{(1-OX)}$	Reported G_{CH_4}
**Sub-total**	**941**	**254**	**398**	**289**				

[a] There is one entry of an open landfill with flaring that reported measured recovery 1300 times higher than modeled generation, which is presumably an error and this value was not considered. There were no other landfills with measured recovery greater than 2 times modeled generation.

[b] For closed landfills, mean values of the ratio of CH_4 that is flared or converted to energy to modeled CH_4 generation are 42% and 58%, respectively, based on the closed landfills that reported a ratio of <95% ratio for R/GCH_4.

[c] For open landfills, mean values of the ratio of CH_4 that is flared or converted to energy to modeled CH_4 generation are 45% and 53%, respectively, based on the open landfills that reported a <90% ratio of R/GCH_4.

Table S12: Summary statistics for collection efficiencies reported by landfills with a gas collection system based on the U.S. Greenhouse Gas Inventory 2021.[1]

	**Closed Landfills**	**Operational Landfills**
**Number of landfills with missing or zero CE**	41 of 183	254 of 941
**1st quartile**	88	64
**Median**	95	74
**Mean**	87	70
**3rd quartile**	95	80
**Max**	95	93

Table S13: Modeled methane generation and measured methane recovery in million metric tons (Mt) and CH_4 collection efficiency (%) by gas collection system type and climate.

Gas Collection System	Climate Region	Measured CH_4 Recovery (Mt)	Eq (S2) for landfills w/o GCS + Eq (S3) for landfills w/ GCS				Eq (S2) for landfills w/o GCS + Eq (S4) for landfills w/ GCS	
			Ratio of CH_4 Recovered to Modeled CH_4 Generated		Annual Modeled CH_4 Generation (Mt)		Estimated Collection Efficiency[d] (%)	Estimated CH_4 Generation (Mt)
			Lower[c]	Upper[c]	Lower[c]	Upper[c]		
**Original Total**[a]					**12.24**			
**No Active GCS**	**Arid**	0	0%	0%	0.18	0.18	0%	0.18
	**Moderate**	0	0%	0%	0.21	0.21	0%	0.21
	**Wet**	0	0%	0%	0.58	0.58	0%	0.58
**Flare**[b]	**Arid**	0.59	48%	53%	1.12	1.24	74%	0.80
	**Moderate**	1.16	50%	61%	1.89	2.19	74%	1.57
	**Wet**	1.62	45%	49%	3.29	3.59	74%	2.19
**Energy Recovery**	**Arid**	0.32	49%	55%	0.58	0.64	74%	0.58
	**Moderate**	1.27	56%	67%	1.89	2.26	77%	1.66
	**Wet**	1.51	50%	54%	2.79	3.00	75%	2.01
**Adjusted Total**		6.47	46%	52%	12.53	14.01	67%	9.64

[a] Sum of raw data that has some data where measured recovery is larger than the modeled generation.

[b] One landfill reported measured recovery 1300 times greater than the modeled generation (i.e., 0.004 Mt) and was removed from the dataset.

[c] Lower and upper estimates for the ratio of recovered to generated CH_4 and annual modeled CH_4 generation correspond to the results adjusted after the corrections in Table 3 for the 68 landfills in which R exceeded GCH_4, 6 landfills with missing recovery data, and 36 landfills that reported a ratio of R to $GCH_4 > 95\%$ or $> 90\%$ for closed and open landfills, respectively.

[d] One closed landfill with a flare did not report the collection efficiency. The mean collection efficiency (86%) of non-zero collecting landfills that are closed and flare their gas was assumed. Collection efficiencies are self-reported.

Table S14: Volumes in million m^3 (Mm^3) of LFG that is uncollected ($V_{LFG\ fugitive}$), flared ($V_{LFG\ flare}$), and converted to energy $V_{LFG\ ICE}$ using Eqs (S3) and (S4).

<table>
<tr><th rowspan="3">Climate Region</th><th colspan="6">Eq (S2) for landfills w/o GCS + Eq (S3) for landfills w/ GCS</th></tr>
<tr><th colspan="2">Mm^3 LFG generated</th><th rowspan="2">Mm^3 LFG flared</th><th rowspan="2">Mm^3 LFG converted to energy</th><th colspan="2">Mm^3 fugitive LFG not reduced for oxdn[a]</th></tr>
<tr><th>Lower</th><th>Upper</th><th>Lower</th><th>Upper</th></tr>
<tr><td>Arid</td><td>6,353</td><td>6,995</td><td>1,994</td><td>1,081</td><td>3,278</td><td>3,920</td></tr>
<tr><td>Moderate</td><td>13,482</td><td>16,118</td><td>3,920</td><td>4,291</td><td>5,271</td><td>7,907</td></tr>
<tr><td>Wet</td><td>22,504</td><td>24,227</td><td>5,474</td><td>5,102</td><td>11,928</td><td>13,651</td></tr>
<tr><td>Total</td><td>42,339</td><td>47,340</td><td>11,387</td><td>10,475</td><td>20,477</td><td>25,478</td></tr>
<tr><th colspan="7">Eq (S2) for landfills w/o GCS + Eq (S4) for landfills w/ GCS</th></tr>
<tr><td>Arid</td><td colspan="2">4,764</td><td>1,994</td><td>1,689</td><td colspan="2">1,689</td></tr>
<tr><td>Moderate</td><td colspan="2">11,624</td><td>3,920</td><td>3,413</td><td colspan="2">3,413</td></tr>
<tr><td>Wet</td><td colspan="2">16,152</td><td>5,474</td><td>5,575</td><td colspan="2">5,575</td></tr>
<tr><td>Total</td><td colspan="2">32,540</td><td>11,387</td><td>10,678</td><td colspan="2">10,678</td></tr>
</table>

5 Figures

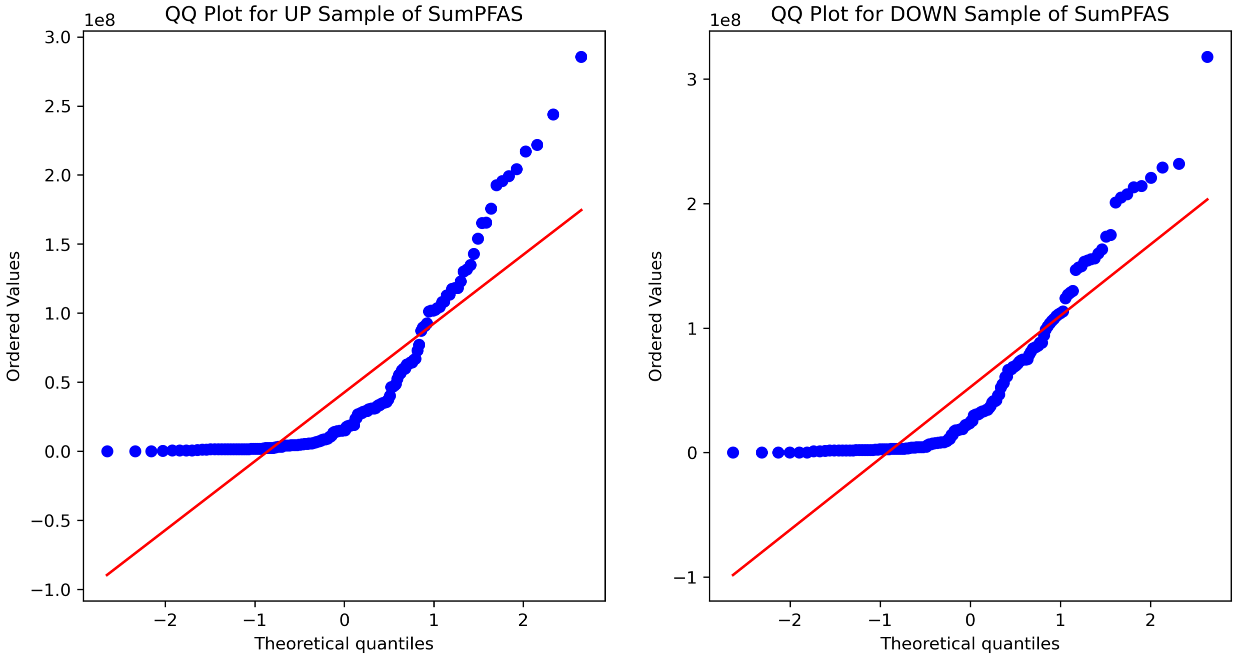

Figure S1: QQ-Plot for SumPFAS concentration upstream (UP) and downstream (DOWN) of the condesate knockout

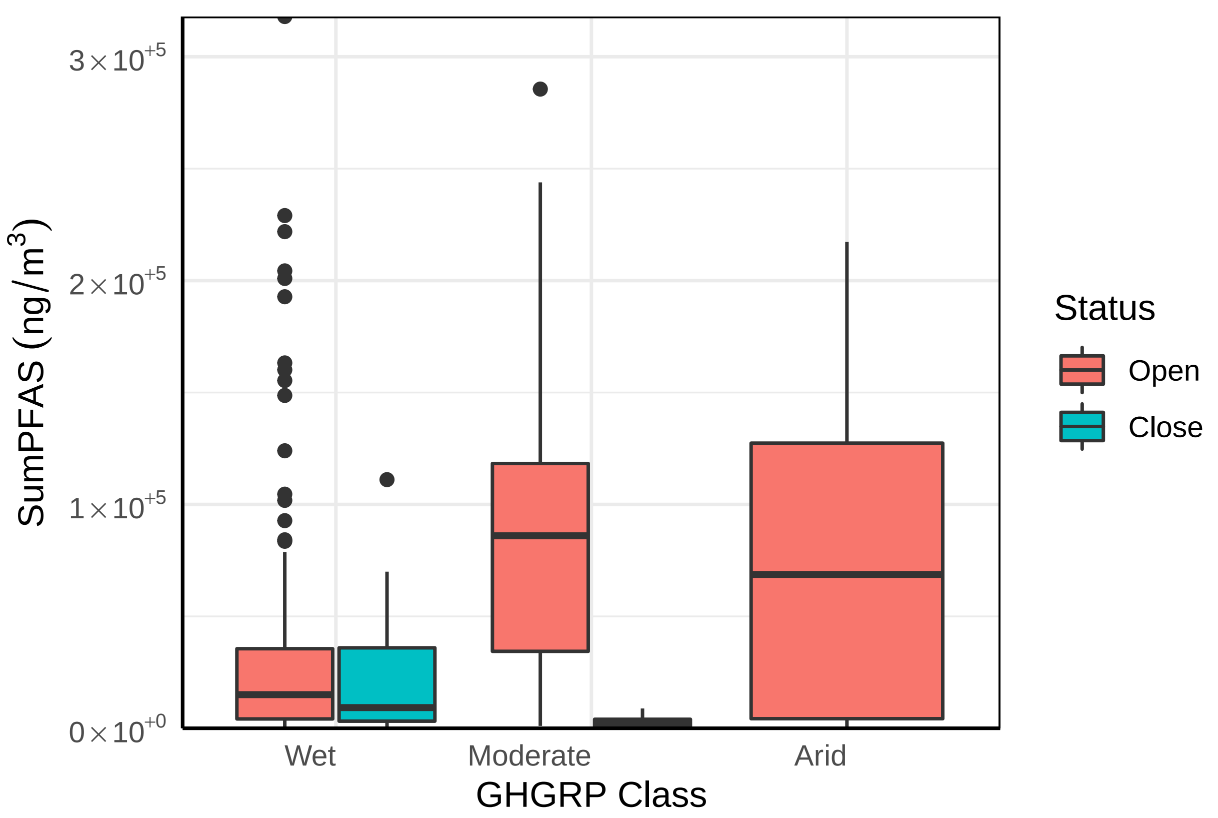

Figure S2: Comparison of SumPFAS from open and closed landfills.

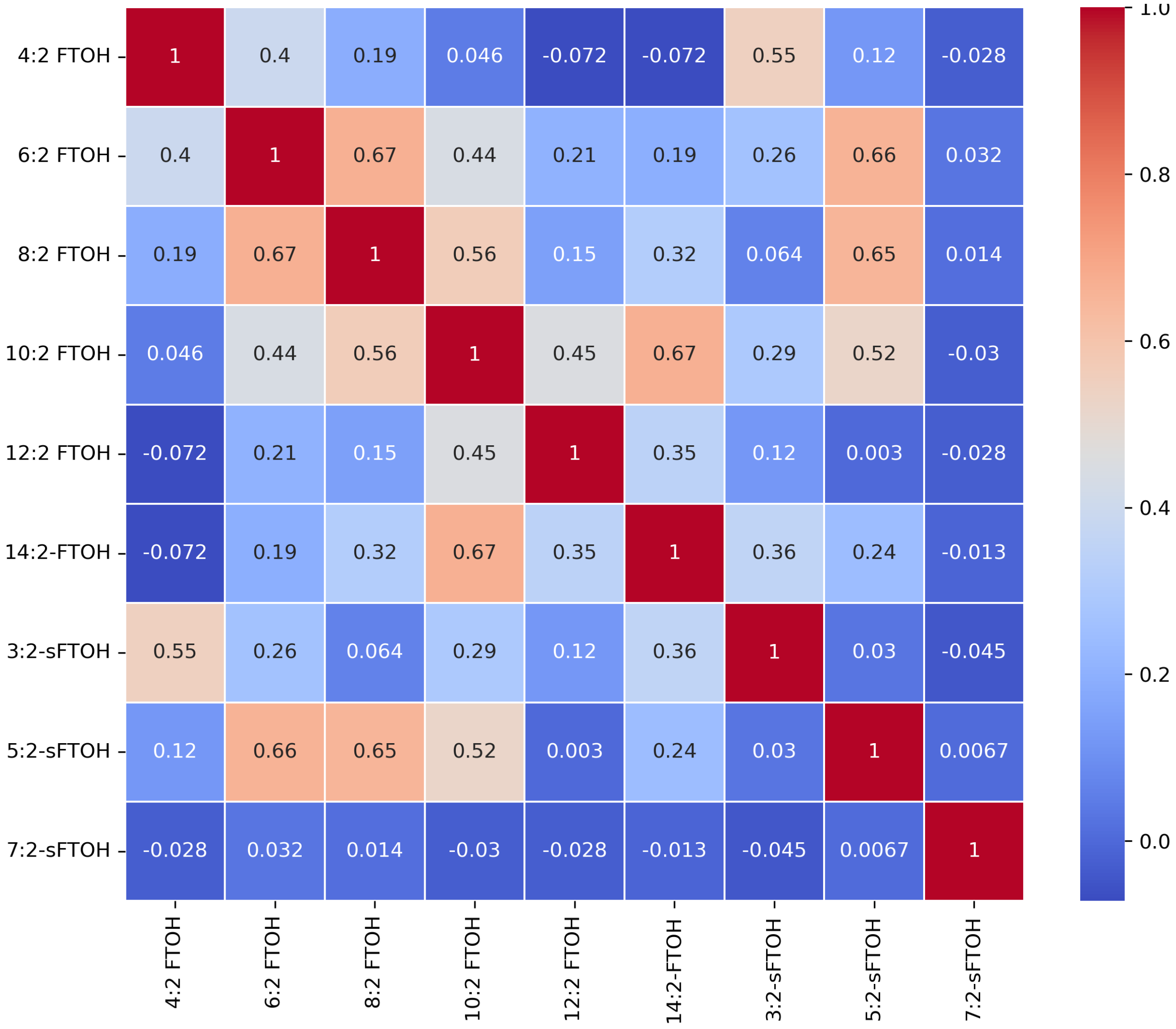

Figure S3: Correlation heat map on the relationship of FTOH and sFTOH.

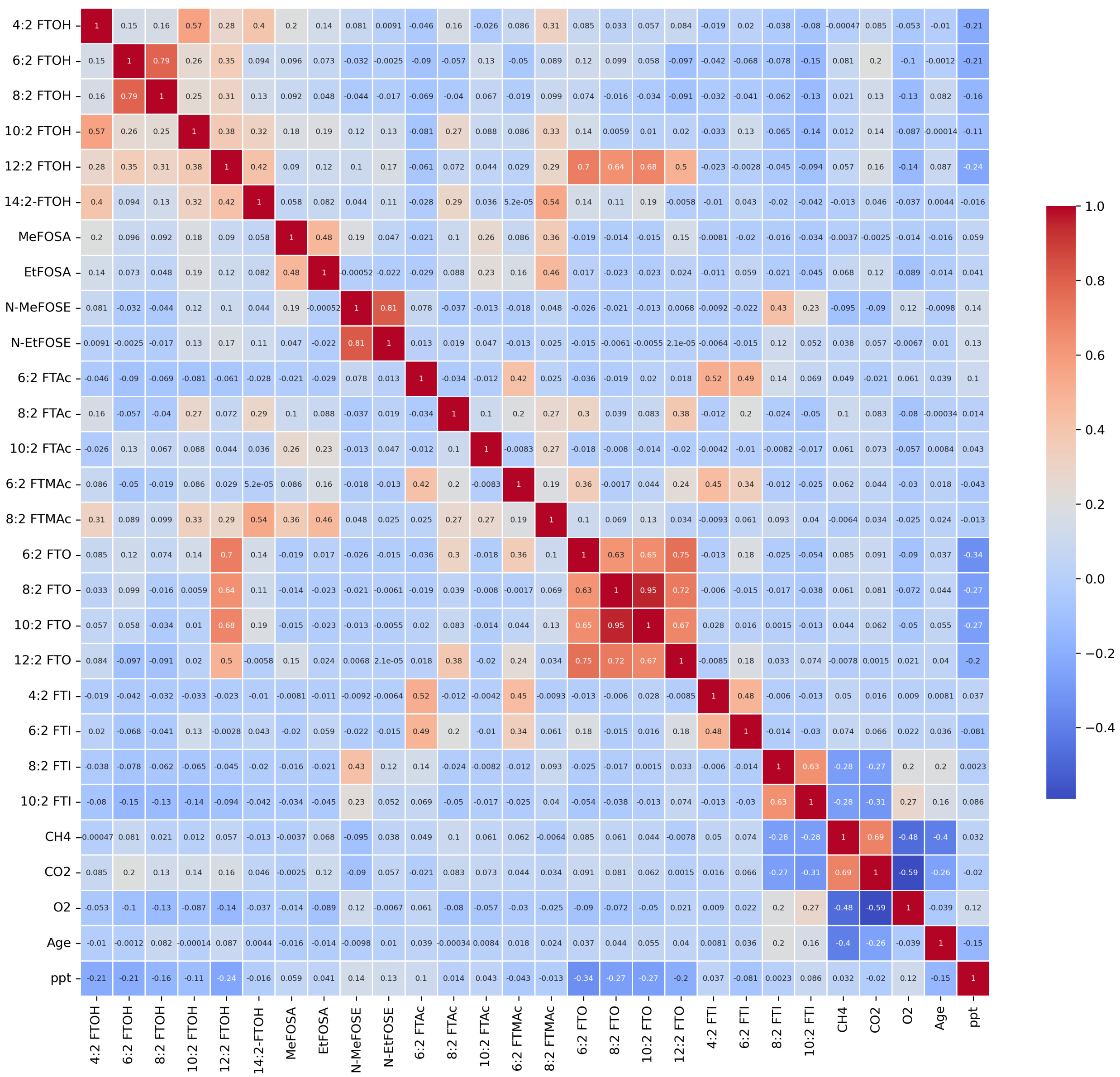

Figure S4: Correlation heat map to evaluate the linear relationships between the PFAS and landfill characteristics

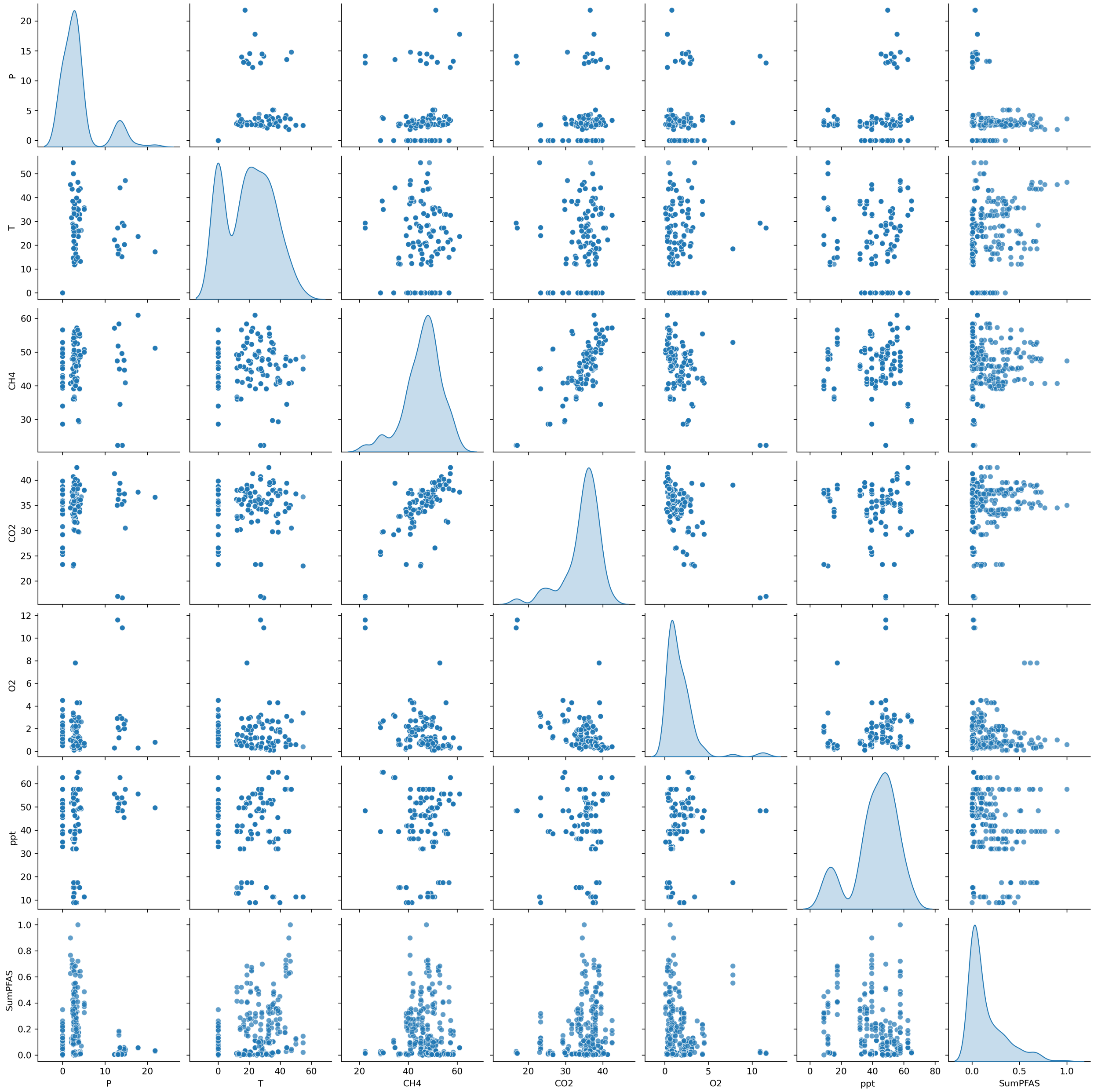

Figure S5: Pairplot showing the inter-relationship between PFAS and site and environmental characteristics

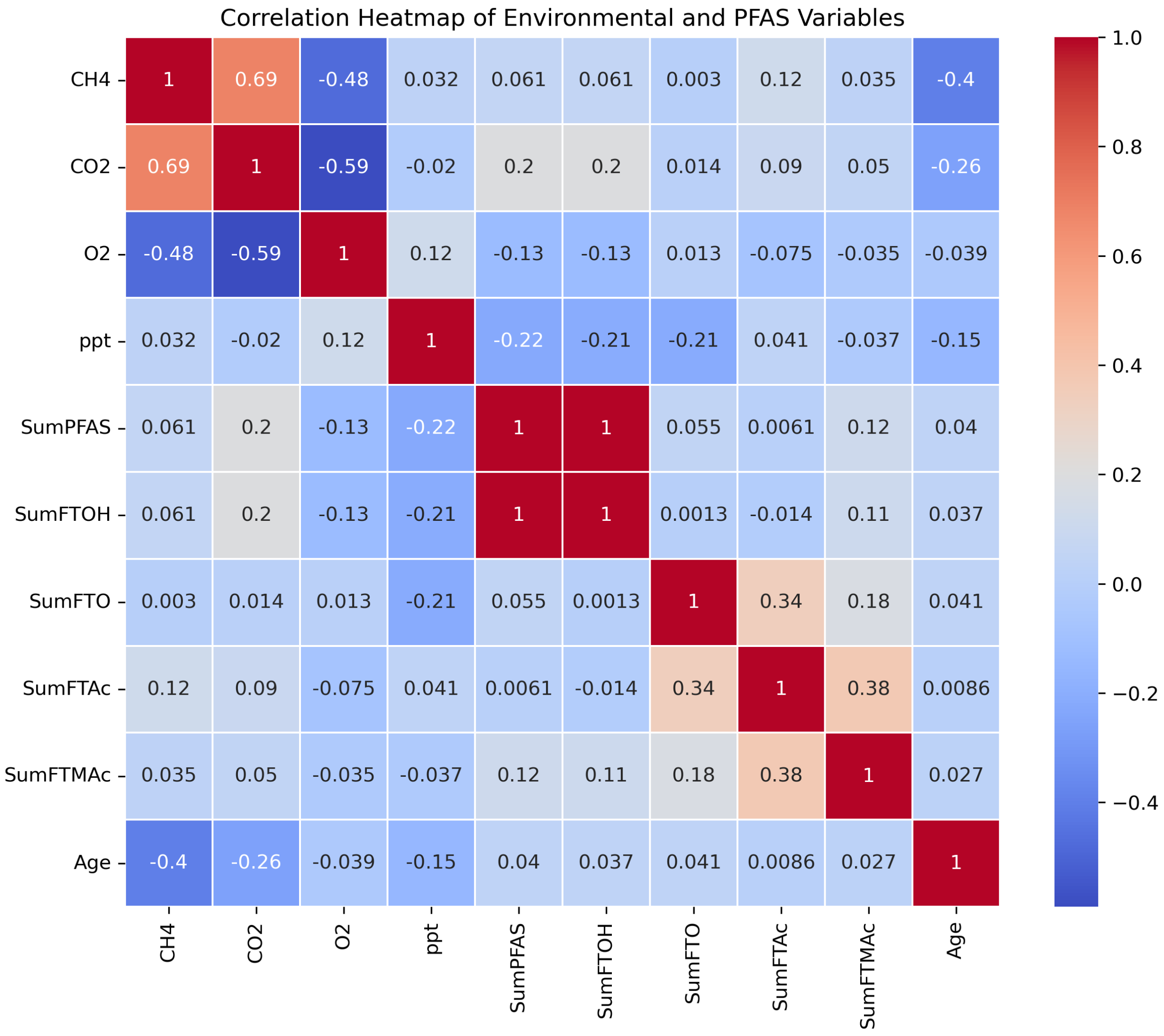

Figure S6: Correlation heat map of PFAS groups and environmental variables.

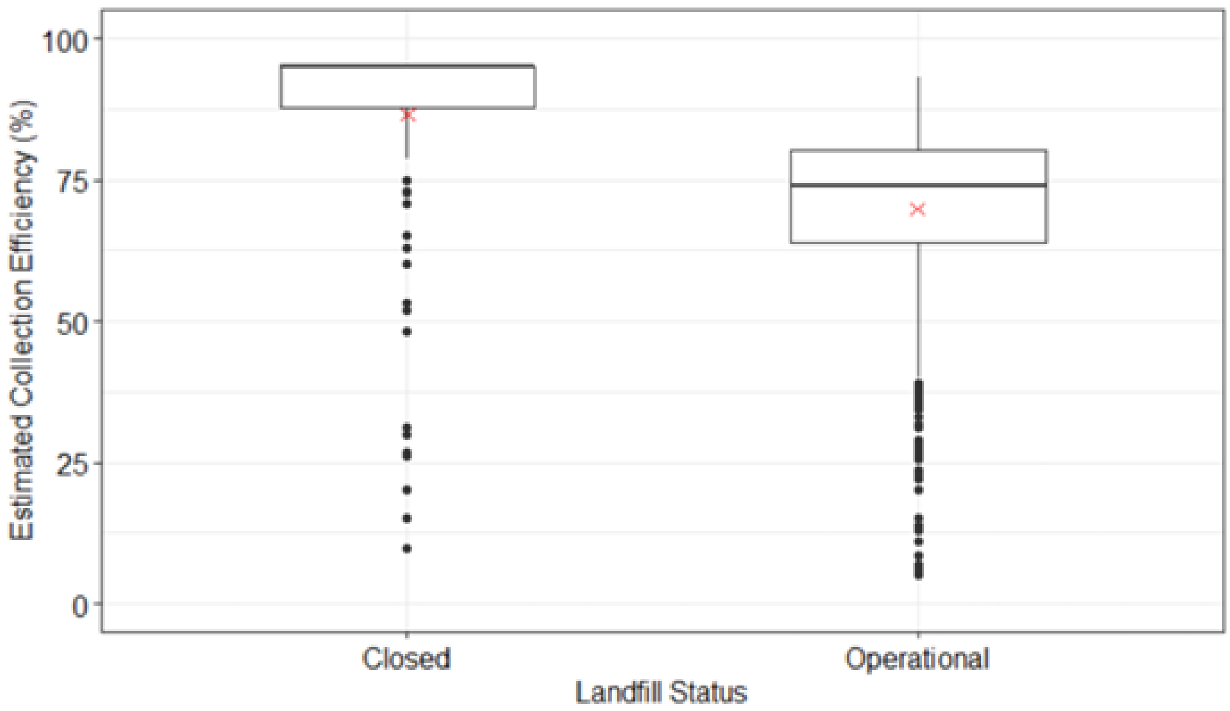

Figure S7: Boxplot showing the assumed collection efficiencies of closed and operational landfill sites in the USGHGI. The × represents the mean of each data set, while the interior line represents the median. The boxes bound the interquartile range (IQR) (25th to 75th quartile). The whiskers extend to 1.5 times the IQR, and their ends show the highest and lowest value excluding outliers, which are represented by solid black points.

References

(1) U S Environmental Protection Agency, *Inventory of U.S. Greenhouse Gas Emissions and Sinks: 1990 – 2020*; 2022; Vol. EPA 430-P-17-001.

(2) U.S. Environmental ProtectionAgency, Landfill Methane Outreach Program. 2019; https://www.epa.gov/lmop/basic-information-about-landfill-gashttps://www.epa.gov/lmop/basic-information-about-landfill-gashttps://www.epa.gov/lmop/basic-information-about-landfill-gas.

(3) U.S.EPA, *AP-42 Emission factors for Municipal Solid Waste Landfills- Supplement E*; 1998; ID: 142.

(4) 40 CFR Part 98 subpart HH., Mandatory Greenhouse Gas Reporting. 2009.

(5) 40 CFR Part 98 subpart HH, Revisions to the Greenhouse Gas Reporting Rule and Final Confidentiality Determinations for New or Substantially Revised Data Elements; Final Rule. 2013.

(6) IPCC, *2019 Refinement to the 2006 IPCC Guidelines for National Greenhouse Gas Inventories*; 2019.

(7) NOAA National Centers for Environmental Information, Climate at a Glance: City Time Series. 2020; https://www.ncdc.noaa.gov/cag/https://www.ncdc.noaa.gov/cag/https://www.ncdc.noaa.gov/cag/.
